# Direct evidence that density-dependent regulation underpins the temporal stability of abundant species in a diverse animal community

**DOI:** 10.1098/rspb.2014.1336

**Published:** 2014-09-22

**Authors:** Peter A. Henderson, Anne E. Magurran

**Affiliations:** 1 Centre for Biological Diversity and Scottish Oceans Institute, School of Biology, University of St Andrews, St Andrews, Fife KY16 8LB, UK; 2 Pisces Conservation Ltd, IRC House, The Square, Pennington, Lymington, Hampshire SO41 8GN, UK

**Keywords:** density-dependence, stability, temporal variability, relative abundance

## Abstract

To understand how ecosystems are structured and stabilized, and to identify when communities are at risk of damage or collapse, we need to know how the abundances of the taxa in the entire assemblage vary over ecologically meaningful timescales. Here, we present an analysis of species temporal variability within a single large vertebrate community. Using an exceptionally complete 33-year monthly time series following the dynamics of 81 species of fishes, we show that the most abundant species are least variable in terms of temporal biomass, because they are under density-dependent (negative feedback) regulation. At the other extreme, a relatively large number of low abundance transient species exhibit the greatest population variability. The high stability of the consistently common high abundance species—a result of density-dependence—is reflected in the observation that they consistently represent over 98% of total fish biomass. This leads to steady ecosystem nutrient and energy flux irrespective of the changes in species number and abundance among the large number of low abundance transient species. While the density-dependence of the core species ensures stability under the existing environmental regime, the pool of transient species may support long-term stability by replacing core species should environmental conditions change.

## 1. Introduction

The quest to explain patterns of biological diversity summarized by species abundance distributions has produced a large literature beginning with Darwin [1] who noted that natural communities contain both common and rare species. Darwin also pointed out that species abundances are not fixed but instead vary through time as a result of competition and changes in the environment. However, the consequences of this temporal turnover for the structure of species abundance distributions remains unclear, in part because of the scarcity of long-term population data covering all the species in a community.

Density-dependence has the potential to act as a strong driver of community dynamics. Although the earliest species abundance models [2,3] did not explicitly consider density-dependence, later models began to include it [4,5]. Chave *et al*. [6], for example, extended neutral theory to take account of density-dependence. It has been assumed [7] that the strength of density-dependence is the same for all species. However, Comita *et al*. [8] reported that, in the Barro Colorado Island forest, rarer species experience stronger density-dependence than the more abundant ones. Johnson *et al*. [9] examined over 150 species of trees within the United States and also found that rare species are subject to more intense density-dependence. At present, then, the perception is either that density-dependence applies equally to all species or is most strongly expressed in rare taxa.

Commonness and rarity of species within a community are linked to their persistence in time. Core species, which are consistently present, are often abundant, whereas transient species occur only occasionally and then usually only in small numbers [10]. Whereas core species are often taxa with adaptations to living in the habitat, transient species may be at the edge of their range or more suited to other environmental conditions. This pattern of occurrence suggests that the temporal dynamics of the two sets of species are shaped by different processes. Biological and statistical factors both play a role in the temporal stability of ecological communities [11–14], but we suggest that their influence depends on whether core or transient species are involved. Specifically, we argue that density-dependence underpins the temporal stability of core species [10]. By contrast, transient species are predicted to have dynamics driven by random environmental events.

Our reasons for predicting that density-dependence plays an important role in the dynamics of the core taxa are twofold. First, Brook & Bradshaw [15] examined over 1000 time series of population abundance and found that the majority showed evidence of density-dependence. This result held across a wide range of taxa. The likelihood of detecting density-dependence increased with the length of the time series as populations for which there are long-term datasets are most likely to show zero net growth (a logical outcome of persistence) [15]. While Brook & Bradshaw examined population data collected from a variety of sources, their finding should also apply to persistent (i.e. core) species within a single community. Such analyses have been impeded by the absence of comprehensive community time series, as noted above. Our second line of evidence is provided by Mutshinda *et al*. [16] who examined the temporal dynamics of the most abundant (and persistent) species in a number of vertebrate and invertebrate communities. Mutshinda *et al*. concluded that while environmental fluctuations are important drivers, density-dependence keeps the populations of dominant species within bounds.

We test our prediction that density-dependence underpins the temporal stability of core species using an estuarine fish community sampled monthly for 33 years. Our tests for density-dependence focus on the numerical abundances of the species in the community. We then examine the community consequences of these temporal dynamics using biomass as our currency; biomass is a direct measure of how the available capacity in a community is allocated among species, and an important community property.

## 2. Material and methods

### (a) Data collection

The ongoing sampling of Bristol Channel estuarine fish assemblage has completed 33 years [17,18]. To date, 81 species and more than 150 000 individuals have been recorded.

Fish samples are collected from the cooling water filter screens at Hinkley Point B Power Station, situated on the southern bank of the Bristol Channel in Somerset, UK (51°14′14.05″ N, 3°8′49.71″ W). The water intakes are in front of a rocky promontory within Bridgwater Bay, and to the east are the 40 km^2^ Stert mud flats. Depending upon the tide, fishes are sampled from water varying in depth from about 8 to 18 m. The filter screens have a solid square mesh of 10 mm. For a full description of the intake configuration and sampling methodology, see [19,20].

Quantitative sampling commenced in 1980 when 24 h surveys of the diurnal pattern of capture were undertaken in October and November. From these surveys, it was concluded that samples collected during daylight were representative of the 24 h catch [21] and monthly quantitative sampling commenced in January 1981. The total volume of water sampled per month, which has not varied over the entire period, is 4.27 × 10^5^ m^3^. To standardize for tidal influence, all sampling dates are chosen for tides halfway between springs and neaps, with sampling commencing at high water (normally about 12.00). Fishes are collected hourly from two filter screens for a 6 h period, identified to species and the number of individuals recorded.

Fish numerical abundance and length has been recorded since the beginning of the survey, and biomass (wet weight) since 2000. Methodology has not changed over the 33 years of study.

### (b) Data analysis

Fish species were classified as either core (i.e. persistent) or transient members of the community. Core species [10] were defined as present in more than 22 of 32 full years analysed (1986 was excluded from the identification of core species as only six months were sampled). As the distribution of persistence is strongly bimodal, with a group of species that are almost always present, and another that occur very infrequently, the precise position of the boundary between persistent and transient species does not affect conclusions about the structure of the community [10]. A small number of taxa (shad, salmon, eel and lamprey species) are passage migrants and move through the study area; these were not considered part of the community and were excluded from the analysis. In the analysis, we used numerical abundance data to test for the presence of density-dependence and examined the consequences of this on community biomass.

Density-dependence is difficult to identify given population abundance data with unknown levels of sampling error. Because the possible methods using ecological time series are subject to both type I and II errors, we used a battery of five methodologies each applied in a conservative manner as follows.

(i) A nonlinear relationship between log population change and log population size, or the presence of a threshold when the relationship abruptly changes provides evidence of density-dependence [22]. We note here that a simple linear negative relationship provides insufficient support for density-dependence because it can be generated by census error. A threshold or nonlinear response, in contrast, is not sensitive to type I error caused by census errors [22].
(ii) Density-dependence is consistent with a log population change versus log population size relationship with a slope steeper than −1. It is important to recognize that a random walk with measurement error generates a gradient of between 0 and −1 and therefore a negative value within this range is not necessarily indicative of density-dependence. However, measurement error acts against the observation of a slope of steeper than −1. Accordingly, a slope steeper than −1 in the presence of measurement error is convincing support for density-dependent regulation and is robust to type I error.
(iii) The R and R* tests of Bulmer [23] are widely used to detect density-dependence and were applied to all time series with no zero annual abundances. Bulmer's R test is sensitive to type I error in the presence of sampling error, whereas R* is not. However, R* lacks power and so is vulnerable to type II error. We therefore used both tests and while we accepted a significant R value as support for density-dependence, we considered the evidence particularly robust when both tests showed significance.
(iv) For the most abundant species (bass, *Dicentrachus labrax*; five-bearded rocking, *Ciliata mustella*; sea snail, *Liparis liparis*; sole, *Solea solea*; sprat, *Sprattus sprattus*) growth and mortality of the age classes present could be followed through time and analyses to detect negative changes in growth, recruitment or survival linked to increased population density could be undertaken. These methods are not based on the analysis of abundance time series and therefore not subject to the same vulnerabilities to type I and II errors.
(v) Species that were regularly unrecorded and never found in large numbers or biomass were considered not to show evidence for density-dependence if their time series could not be statistically distinguished from a random time series, or if their occurrences were too infrequent to allow any test.

## 3. Results

Of the 27 core fish species present, 23 show evidence of density-dependence and the remaining four core species do not. All the transient species have time series that cannot be distinguished from random noise or are too rare to be fitted to any model.

**Figure 1.**
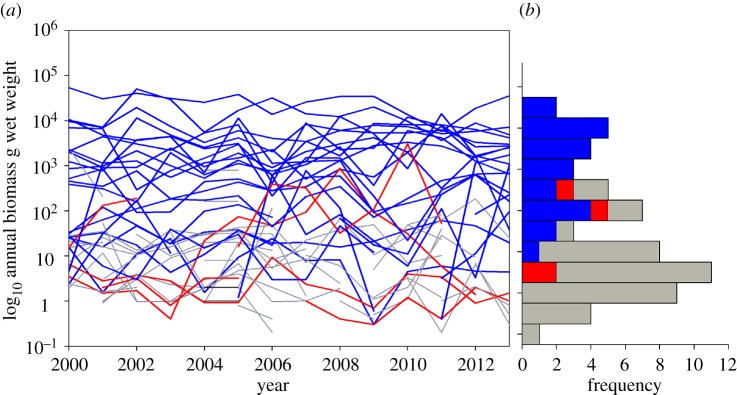
Temporal variability within the fish community of Bridgwater Bay. (*a*) Variation in annual biomass. Core species showing density-dependence are shown in blue, core species with no evidence of density-dependence in red, and transient species in grey. (*b*) The frequency distribution of average abundance over the 12-year period.

Temporal variation in biomass is muted in all core species showing density-dependence relative to those that do not (figure 1*a*). These species predominate at the common end of the species abundance distribution, whereas core species that are not experiencing density-dependence are found in the middle region of the species abundance distribution, and transient species cluster at the rare end (figure 1*b*).

**Figure 2.**
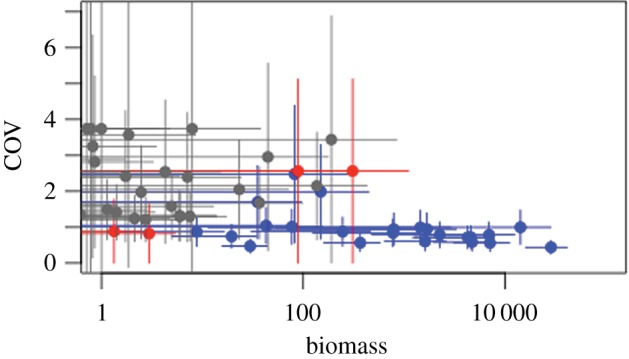
Relationship between COV (±s.d.) and mean (±s.d.) biomass. Core species showing density-dependence are shown in blue. Core species with no evidence of density-dependence in red. Transient species in grey.

The difference in temporal variability of the core density-dependent, core non-density-dependent and transient species is highlighted in figure 2, which plots the coefficient of variance (COV) in biomass against the mean biomass. What is striking is the high degree of uncertainty associated with transient species; core species that are not density-dependent also exhibit this pattern. By contrast, core species experiencing density-dependence show little temporal movement. The temporal stability of the biomasses of this group of persistent fishes is evident from the fact that the top right-hand portion of figure 2 is empty.

## 4. Discussion

A major task in ecology today is understanding how biodiversity stabilizes assemblages [24]. Our results show that species make different contributions to the stability of a community and underline the role that density-dependence plays. Species experiencing density-dependence exhibit relatively little temporal variation in biomass. These species also typically account for a large fraction of the overall abundance; in the case of the Hinkley Point assemblage, they represent more than 98% of total biomass. Because of the stability in biomass of these core species, it is inevitable that nutrient and energy flux will also be stabilized.

Temporal variation in population abundance can enable communities to persist [24,25]. Of particular interest is the idea that populations fluctuate asynchronously [26] because asynchronicity can stabilize community properties. Seasonal and annual fluctuations in abundance [27] among the core species in the Hinkley Point community enable assemblage members to coexist while competing along a limited number of resources axes. However, as we show here, it is not simply asynchronicity in species abundances that helps maintain community properties. Density-dependent processes acting on the core species also contribute to stability in biomass production, for example.

This community is made up of species belonging to distinct spatial guilds that include pelagic taxa, those that live on rocky surfaces and those associated with soft or weedy bottoms [28]. In each case, the dominant species in these spatial guilds [28] shows density-dependence indicating that species associated with different habitats contribute to the stability of the community as a whole.

As we have demonstrated, species that are density-dependent tend not just to be common but also to be consistently common. This means that these species stabilize the species abundance distribution at its high abundance end. Understanding the dynamics of the component species therefore provides important clues about the stability of the assemblage as a whole [29]. Moreover, better knowledge of how these density-dependent processes respond to external drivers, including anthropogenic change, may improve predictions about the fate of natural systems in a rapidly changing world.

What are the implications of these results for the shape of the species abundance distribution? Engen [30] modelled distributions in which species varied in the strength of negative feedback control (density-dependence) and environmental noise experienced. Despite the heterogeneous nature of these models, the species abundance distributions generated resembled lognormal and gamma models common in natural communities [31]. It is therefore unsurprising that an extensive analysis of Australian bird communities [32] found no relationship between the shape of the species abundance distribution and ecological variables. Nonetheless, the shape of a species abundance distribution is influenced by temporal events which only become clear once the population dynamics of the individual species in the assemblage are considered [33].

Our results reveal that density-dependence is not uniform across species, though in contrast to Comita *et al*. [8] we find it operating in common species rather than rare ones. However, in the Hinkley Point system, common species are those that are persistent, and while they may have relatively high biomass this need not imply high numerical abundance. For example, the conger eel ranks second in biomass but only 20th in numerical abundance. In terms of number of individuals per unit area of seabed, the conger eel would be termed rare but is under density-dependent control. The difference in view may arise in part because of the use of spatial density rather than biomass to define rarity for trees. Another contributing factor may be that trees are often long-lived. Trees can be rare for a long time if only a few individuals in a species become established, with strong density-dependence ensuring that the population does not grow.

An additional reason for the different conclusions reached about the role of density-dependence in this marine system, relative to tree assemblages, is that the rare (transient) species we observed may be subject to density-dependent mechanisms elsewhere, but because they are near the edge of their range, or accidental visitors to unsuitable habitat, their abundances at Hinkley Point vary at random. As transients, these species are unlikely to be affected by ecological interactions in this assemblage [34]. It is also worth noting that marine systems often have higher levels of β diversity [35] than terrestrial ones, an observation consistent with higher rates of temporal turnover. Rapid temporal turnover will reduce the scope for density-dependence to operate on rare species in these systems.

We hypothesize that natural communities are hybrids of two dynamical behaviours, a core group of persistent species, often dominant in biomass terms, which display negative feedback dynamics and muted variability, and a larger group of transient species displaying quasi-random abundance as they are unable to establish permanent residency. This idea is consistent with recent research [36] highlighting differences in the processes that shape the abundances of the numerical dominant species versus rare species in marine ecosystems. The two groups of species make different contributions to community stability. In the case of core species, abundances are limited by carrying capacity and the resultant density-dependent dynamics help ensure that biomass production is maintained. The transient species abundances, on the other hand, are influenced by environmental conditions, are not generally constrained by resource availability and therefore do not exhibit density-dependent dynamics. However, it is these transient species that account for most of the species richness. Moreover, even though they are currently infrequent, it is the transient species that have the potential to increase in abundance should environmental conditions change, and on which the longer term stability of the system depends.

## Supplementary Material

Summary of results of density-dependence tests

## Supplementary Material

Figure ESM1

## Supplementary Material

Notes on detailed calculations

## Data accessibility

A summary of the density-dependence test results, including information on the detailed analyses on abundant species is given in the electronic supplementary material; annual abundance data are archived at doi:10.5061/dryad.3090c.
