# Supplementary material for: Direct evidence that density-dependent regulation underpins the temporal stability of abundant species in a diverse animal community
Source: Proc Biol Sci. 2014 Sep 22;281(1791):20141336. doi: 10.1098/rspb.2014.1336 (PMC4132688; doi:10.1098/rspb.2014.1336)
Supplement: Summary of results of density-dependence tests [file rspb20141336supp1.pdf]

	Species	Total number recorded 1981-2012	Guild	Years present	Core species	Density Dependence detected	Non-linearity /threshold in log pop.- Log pop change plot	log pop. /Log pop. change gradient > -1	Bulmers R	Bulmers R*	Notes on detailed analysis
1	Sprattus sprattus (L.) - Sprat	46734	Pelagic	31	Yes	Yes	No	No	Sig. 5% level	No	Recruitment declines with increasing adult abundance giving strong evidence for density-dependence. Stock-recruitment relationship fits a Ricker curve.
2	Merlangius merlangus (L.) - Whiting	43241	Proximo-benthic	31	Yes	Yes	Yes	Yes- when abundance above threshold	Sig. 5% level	Sig. 10% level	
3	Pomatoschistus minutus (Pallas)-sand goby	9579	soft benthic	31	Yes	Yes	No	No	Sig. 5% level	Sig. 5% level	
4	Solea solea L. - Dover sole	7235	soft benthic	31	Yes	Yes	No	No	Sig 5% level	No	Mortality increases with recruitment - population number has followed a sigmoidal curve and is now varying around carrying capacity
5	Trisopterus minutus (L.) - poor cod	6296	Proximo-benthic	30	Yes	Yes			Sig. 5% level	Sig. 5% level	
6	Trisopterus luscus (L.) - pout	3970	Proximo-benthic	31	Yes	Yes	No	No	Sig. 5% level	Sig. 5% level	Pout abundance notably stable with no trend
7	Liparis liparis (L.) - sea snail	2865	soft benthic	31	Yes	Yes	No	No	Sig. 5% level	Sig. 5% level	Published work Henderson, P. A. & Seaby, R. M. H. (1999) shows a high proportion of temporal variability linked to temperature mediated migrations. Underlying population remarkably stable.
8	Platichthys flesus (L.)- flounder	2862	soft benthic	31	Yes	Yes	Yes	Yes- when abundance above threshold	Sig. 5% level	No	
9	Clupea harengus L. - herring	2688	Pelagic	29	Yes	Yes	No	No	Sig 5% level	No	Population recovering with an almost steped increase in abundance post 2000
10	Dicentrarchus labrax (L.) - bass	2599	Proximo-benthic	31	Yes	Yes	No	No	Yes	No	Published work Henderson and Corp showing that high recruitment suppresses subsequent recruitment for next 2 years.
11	Ciliata mustela (L.) -5-bearded rockling	2061	soft benthic	31	Yes	Yes	No	No	Sig. 5% level	No	Recently the exponential increase has stabilised and now the over-winter loss of biomass is greatest in high biomass years giving good evidence for density-dependence.
12	Gadus morhua L. - cod	1781	Proximo-benthic	30	Yes	Yes	No	Yes but -1.095 so unconvincing	Sig. 5% sig level for both number and biomass	Sig. 5% for biomass	Biomass notably stable with no long-term trend
13	Limanda limanda (L.) - dab	1579	soft benthic	31	Yes	Yes	Yes	Yes when data is split pre and post 1994	Sig. 5% level	No	Abundance is temperature dependent which is why the data set shows discontinuity
14	Liza ramada (Risso) - thin-lipped mullet	1516	sheltered	31	Yes	Yes	No	No	Sig. 5% level	Sig. 5% level	Biomass notably stable with no long-term trend
15	Entelurus aequoreus (L.) - snake pipefish	739	Pelagic	29	Yes	No	No	No	No	No	There was a single large burst in abundance centred on 2008
16	Eutrigla gurnardus (L.) - grey gurnard	518	soft benthic	27	Yes	Yes	No	No	Sig. 5% level	Sig. 5% level	There was a single large burst in abundance centred on 2007
17	Alosa fallax (Lacepede) - twaite shad	516	passage	28	Yes	not included					Passage migrant
18	Aphia minuta (Risso) - transparent goby	445	Pelagic	31	Yes	Yes	No	Yes post 1989 when numbers increased	Sig. 5% level	No	
19	Anguilla anguilla (L.) - eel	315	passage	30	Yes	not included	No	no	No	No	Exponential decline reported Henderson et al (2012) - anthropogenic impact
20	Conger conger L. - conger eel	289	hard benthic	31	Yes	Yes	No	No	Sig. 5% level	Yes 10% sig level	
21	Trisopterus esmarkii - norway pout	240	Proximo-benthic	28	Yes	Yes	No	No	Sig. 5% level	No	
22	Merluccius merluccius (L.) - hake	197	Proximo-benthic	17	No	No			Zero abundances		Species disappeared in 1999 probably linked to climate change.
23	Agonus cataphractus (L.)	191	hard benthic	31	Yes	Yes	No	No	Sig. 5% level	Sig. 5% level	Hooknose abundance has changed little over 30 years
24	Pleuronectes platessa L.	179	soft benthic	28	Yes	Yes	No	Yes	Sig. 5% level	No	
25	Pollachius pollachius (L.)	113	Proximo-benthic	27	Yes	Yes	No	Yes	Sig 5% level	No	
26	Mullus surmuletus L.	111	soft benthic	17	No	No	No	No	Frequent zero abundances		Warm water migrant
27	Cyclopterus lumpus L. -lumpsucker	109	hard benthic	18	No	No	No	No	Frequent zero abundances		Cold water species almost absent siince 2000
29	Ciliata septentrionalis (Collet)	87	soft benthic	28	Yes	Yes	No	No	Sig. 5% level	No	Data from 1999-2011 used for Buller's test. Occurs in small numbers during winter
30	Callionymus lyra L. - dragonette	86	hard benthic	26	Yes	No	No	No	Frequent zero abundances		Too uncommon for analysis, but numbers are notably constant through time.
31	Raja clavata L. - thornback ray	76	soft benthic	26	Yes	No	No	No	Frequent zero abundances		Too uncommon for analysis, but numbers are notably constant through time.
32	Micromesistius poutassou - blue whiting	67	Proximo-benthic	9	No	No					Rare transient:Fits random model
33	Syngnathus rostellatus Nillson	66	sheltered	25	Yes	No	No	No	Frequent zero abundances		Low numbers recorded, but abundance notably constant
34	Maurolicus muelleri (Gmelin) -pearlsides	56	Pelagic	17	No	No	No	No			Deep water transient - fits random model
35	Pomatoschistus microps (Kroyer) - common goby	53	soft benthic	9	No	No			zero abundances		Occassional visitor prefers upper estuarine waters - fits random model
36	Scyliorhinus caniculus (L.) - dogfish	53	soft benthic	23	Yes	Yes	No	No	Sig. 5% level		Numbers are remarkably constant, but small. Analysis undertaken on time series from 1999.
37	Gasterosteus aculeatus L. 3-spined stickleback	52	freshwater	22	No	No	No	No	Zero abundances		Freshwater species which washes out of rivers in winter
38	Syngnathus acus (L.) - greater pipefish	44	sheltered	13	No	No	No	No	Zero abundances		Fits random model
39	Chelidonichthys lucerna L. - tub gurnard	39	soft benthic	15	No	No	No	No	Zero abundances		Fits random model
40	Atherina boyeri Risso - sandsmelt	27	sheltered	14	No	No	No	No	Zero abundances		Lives in lagoons - fits random model
41	Gobius niger L. - black goby	27	soft benthic	11	No	No	No	No	Zero abundances		Fits random model
42	Psetta maxima (L.) - turbot	27	soft benthic	11	No	No	No	No	Zero abundances		Fits random model
43	Liza aurita - golden grey mullet	24	sheltered	4	No	Rare visitor					To rare to fit any model
44	Ammodytes tobianus L. - greater sandeel	22	soft benthic	15	No	No	No	No	Zero abundances		Prefers clean sand -fits random model
45	Lophius piscatorius L. - angler fish	19	soft benthic	7	No	Rare visitor					Deeper water species - fits random model
46	Scophthalmus rhombus (L.) - brill	19	soft benthic	9	No	Rare visitor					Fits random model
47	Trachurus trachurus (L.) - horse mackerel	16	Pelagic	13	No	No	No	No	Zero abundances		Fits random model
48	Labrus bergylta Ascanius - ballan wrasse	15	hard benthic	6	No	Rare visitor					Fits random model
49	Gaidropsaurus vulgaris (Cloquet)-3bearded rockling	10	soft benthic	5	No	Rare visitor					Fits random model
50	Crenimugil labrosus (Risso) - thick-lipped mullet	9	sheltered	4	No	Rare visitor					Fits random model
51	Lampetra fluviatalis (L.) - river lamprey	9	passage	8	No	not included					Passage migrant
52	Salmo salar L. - salmon	9	passage	8	No	not included					Passage migrant
53	Crystallogobius linearis (von Duben) - crystal goby	8	Pelagic	4	No	Rare visitor					Fits random model
54	Hyperoplus lanceolatus (Lesauvage) - greater sandeel	8	soft benthic	5	No	Rare visitor					Fits random model
55	Spinachia spinachia (L.) - 15-spined stickleback	7	hard benthic	6	No	Rare visitor					Fits random model
56	Spondyliosoma cantharus (L.) -black sea bream	7	hard benthic	7	No	Rare visitor					Fits random model
57	Parablennius gattorugine L. -tompot blenny	6	hard benthic	6	No	Rare visitor					Fits random model
58	Ctenolabrus rupestris (L.)- goldsinny	6	hard benthic	5	No	Rare visitor					Fits random model
59	Engraulis encrasicolus (L.) - anchovy	6	Pelagic	3	No	Rare visitor					Fits random model
60	Gobius paganellus - rock goby	5	soft benthic	2	No	Rare visitor					Too rare to fit any model
61	Sardina pilchardus (Walbaum) - pilchard	5	Pelagic	2	No	Rare visitor					Too rare to fit any model
62	Raniceps raninus (L.) - tadpole fish	4	soft benthic	4	No	Rare visitor					Too rare to fit any model
63	Buglossidium luteum (Risso)- solonette	3	soft benthic	2	No	Rare visitor					Too rare to fit any model
64	Centrolabrus exoletus (L.)-goldsinny	3	hard benthic	2	No	Rare visitor					Too rare to fit any model
65	Labrus mixtus - cuckoo wrasse	3	hard benthic	3	No	Rare visitor					Too rare to fit any model
66	Microstomus kitt-lemon sole	3	soft benthic	3	No	Rare visitor					Too rare to fit any model
67	Molva molva (L.) - ling	3	soft benthic	3	No	Rare visitor					Too rare to fit any model
68	Trigla lyra - piper	3	soft benthic	2	No	Rare visitor					Too rare to fit any model
69	Balistes carolinenis (Gmelin) - trigger fish	2	hard benthic	2	No	Rare visitor					Too rare to fit any model
70	Crenilabrus melops (L.) corkwing wrasse	2	hard benthic	2	No	Rare visitor					Too rare to fit any model
71	Petromyzon marinus - sea lamprey	2	passage	2	No	not included					Passage migrant
72	Raja brachyura - blond ray	2	soft benthic	2	No	Rare visitor					Too rare to fit any model
73	Raja microocellata -small eyed ray	2	soft benthic	2	No	Rare visitor					Too rare to fit any model
74	Zeus faber L. - John dory	2	Pelagic	2	No	Rare visitor					Too rare to fit any model
75	Belone bellone (L.) -garfish	1	Pelagic	1	No	Rare visitor					Too rare to fit any model
76	Glyptocephalus cynoglossus -witch	1	soft benthic	1	No	Rare visitor					Too rare to fit any model
77	Nerophis lumbriciformis - worm pipefish	1	sheltered	1	No	Rare visitor					Too rare to fit any model
78	Pollachius virens (L.) - saith	1	Proximo-benthic	1	No	Rare visitor					Too rare to fit any model
79	Trachinus vipera Cuvier - weaver	1	soft benthic	2	No	Rare visitor					Too rare to fit any model
80	Zeugopterus punctatus (Bloch) -topknot	1	hard benthic	1	No	Rare visitor					Too rare to fit any model
81	Ammodytes marinus - sandeel	1	soft benthic	1	No	Rare visitor					Too rare to fit any model
