# Supplementary figures and images for: Direct evidence that density-dependent regulation underpins the temporal stability of abundant species in a diverse animal community
Source: Proc Biol Sci. 2014 Sep 22;281(1791):20141336. doi: 10.1098/rspb.2014.1336 (PMC4132688; doi:10.1098/rspb.2014.1336)

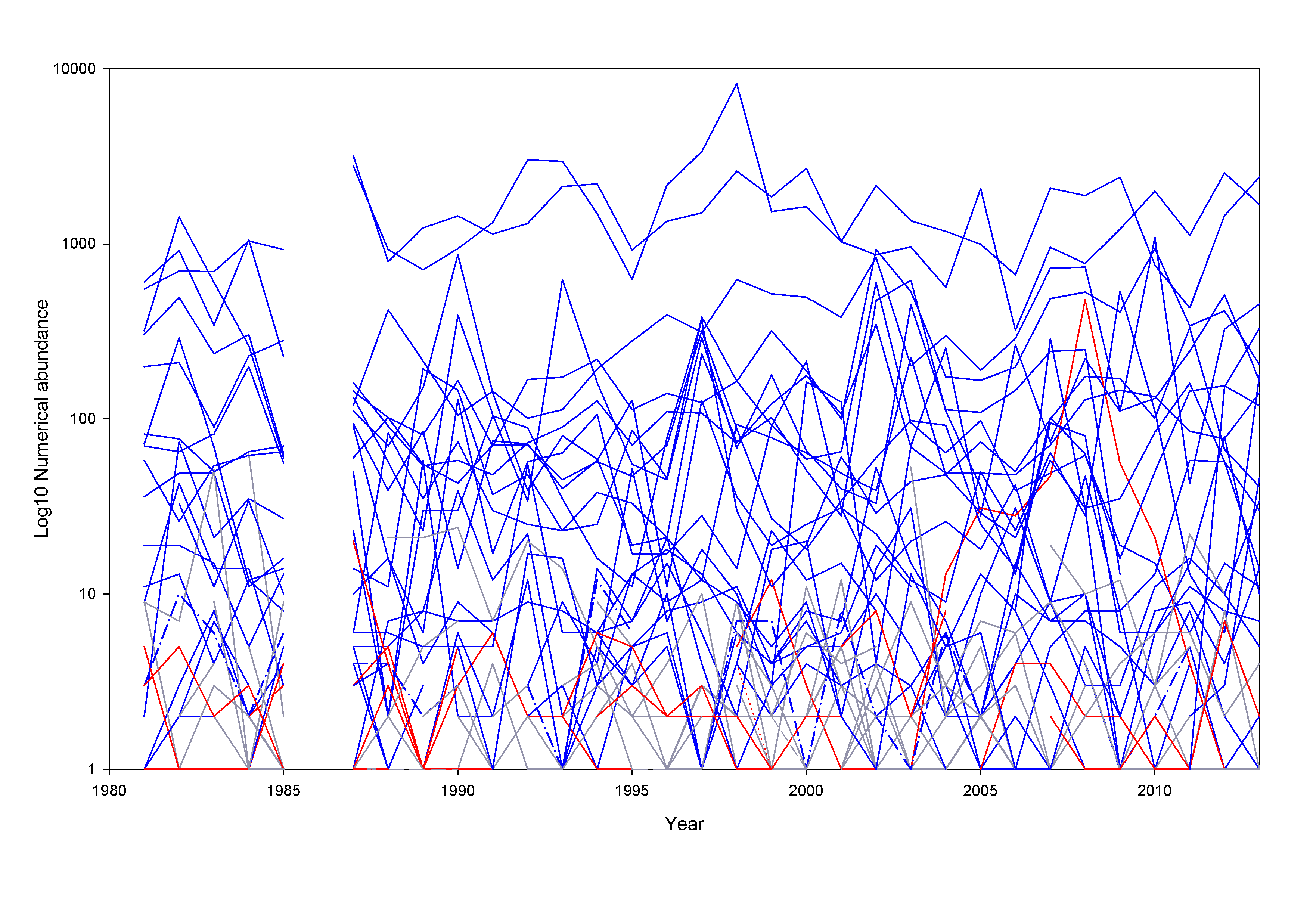

Supplement: Figure ESM1 [file rspb20141336supp2.BMP]
