# Supplementary material for: Direct evidence that density-dependent regulation underpins the temporal stability of abundant species in a diverse animal community
Source: Proc Biol Sci. 2014 Sep 22;281(1791):20141336. doi: 10.1098/rspb.2014.1336 (PMC4132688; doi:10.1098/rspb.2014.1336)
Supplement: Notes on detailed calculations [file rspb20141336supp3.docx]

# Notes on the Detection of Density-Dependence of Individual Fish Species Sampled from Hinkley Point (Bridgwater Bay, Bristol Channel UK) 1981-2013

## Methods

Density-dependence is difficult to identify given population abundance data with unknown levels of sampling error. Because the possible methods using ecological time series are subject to both type I and II errors we used a battery of 5 methodologies each applied in a conservative manner as follows. (1) A non-linear relationship between log population change and log population size, or the presence of a threshold when the relationship abruptly changes provides evidence of density-dependence [[1](#_ENREF_1)]. We note here that a simple linear negative relationship provides insufficient support for density-dependence because it can be generated by census error. A threshold or non-linear response, in contrast, is not sensitive to type I error caused by census errors [[2](#_ENREF_2)]. (2) Density-dependence is consistent with a log population change - log population size relationship with a slope steeper than -1. It is important to recognise that a random walk with measurement error generates a gradient of between 0 and -1 and therefore a negative value within this range is not necessarily indicative of density-dependence. However, measurement error acts against the observation of a slope of steeper than -1. Accordingly, a slope steeper than -1 in the presence of measurement error is convincing support for density-dependent regulation and is robust to type I error. (3) The R and R* tests of Bulmer [[3](#_ENREF_3)] are widely used to detect density-dependence and were applied to all time series with no zero annual abundances. Bulmer’s R test is sensitive to type I error in the presence of sampling error, while R* is not. However, R* lacks power and so is vulnerable to type II error. We therefore used both tests and while we accepted a significant R value as support for density-dependence we considered the evidence particularly robust when both tests showed significance. (4) For the most abundant species, growth and mortality of the age classes present could be followed through time and analyses to detect negative changes in growth, recruitment or survival linked to increased population density could be undertaken. These methods are not based on the analysis of abundance time series and therefore not subject to the same vulnerabilities to type I and II errors. (5) Species that were regularly unrecorded and never found in large numbers or biomass were considered not to show evidence for density-dependence if their time series could not be statistically distinguished from a random time series, or if their occurrences too infrequent to allow any test.

### The Chow Test for Structural Breaks

The Chow test was used to test if the data should be divided by a break point to generate two separate regressions. The significance of the breakpoint or threshold was determined by comparing the results of the two subset regressions versus the regression generated from all the data. The test was undertaken using R and the code is given in Appendix 1 below.

# Results

All of the core fish species in the Bridgwater Bay system are considered in alphabetical order below.

## Bass, *Dicentrachus labrax*

An abundant species in southern British waters that uses estuarine habitat for the first 3 years of life before moving further offshore or to deeper rocky habitat.

### Relationship between log population change and log population size

The slope of log change in population size offers no proof for density-dependence. While the slope is negative, the gradient of -0.5 is less than -1.0. There is also no evidence of a non-linear relationship or a break-point indicating a threshold response.

**The relationship between Log Population Change and Log Population size gives no support for the existence of density-dependence.**


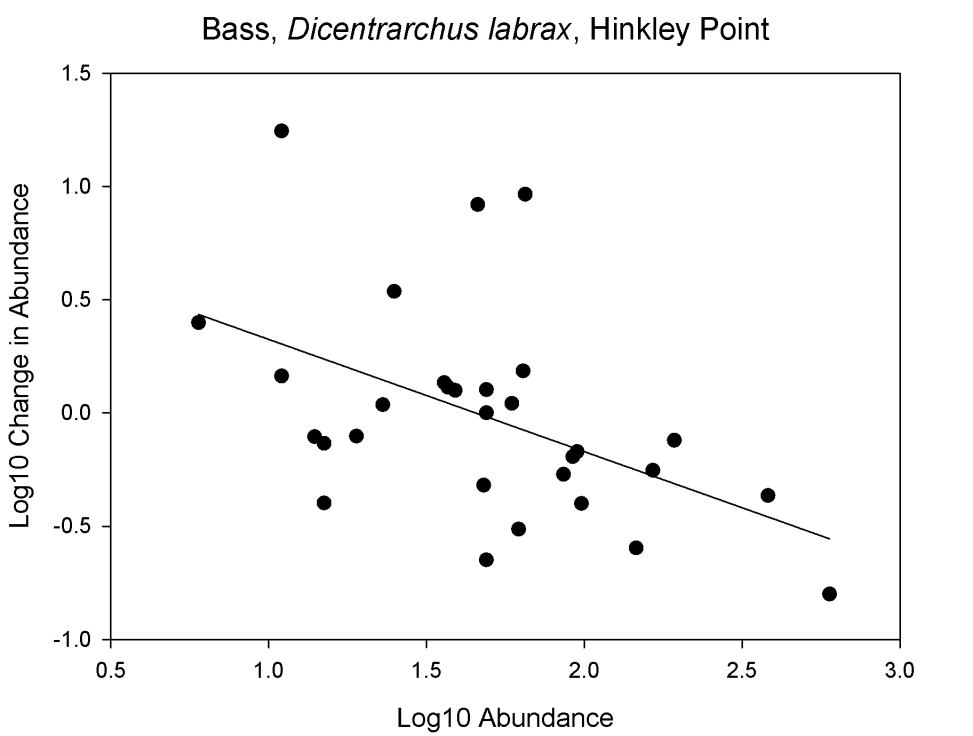


Figure 1 The relationship between population size and change for the bass *Dicentrarchus labrax* at Hinkley Point.

### Bulmer’s test

Using count data from 1987 to 2013 inclusive the results for Bulmer’s tests were as follows:

R = 1.03, R_0.5_ = 1.165, **significant for density dependence at 5% level.**

R^*^= -0.19, R_0.5_ = -0.348, **non-significant for density dependence at 5% level.**

### Additional evidence

Henderson & Corps [[4](#_ENREF_4)] demonstrated a powerful temperature effect on bass abundance and showed a density-dependent suppression of new recruits by 1 and 2 year old fish present in the nursery.

## Cod, *Gadus morha*

Almost all the cod are 0-group and a single cohort is present from June through to the following March. To follow year class strength the abundance summed over this period is used to estimate the year class abundance. In the early 1980s there were almost no cod present so the time series analysis can only start from 1986 when no more zeros were observed.

Examination of cod shows a population showing pulsed recruitment inputs which gradually decline as the O-group die off. An odd feature is that the pulses seem to be getting greater in magnitude through time.

### Relationship between log population change and log population size

The relationship between log population change and log population size has a negative slope (-0.7 for a linear regression fit, R^2^ =0.36) but is a poor fit to a straight line (Figure 2). The plot shows a fit to a quadratic, although there is no biological support for the use of this function it demonstrates that a curve gives a marginally better fit (R^2^ =0.39).

**Non-linear relationship between Log Population Change and Log Population size producing a better fit indicates density-dependence.**


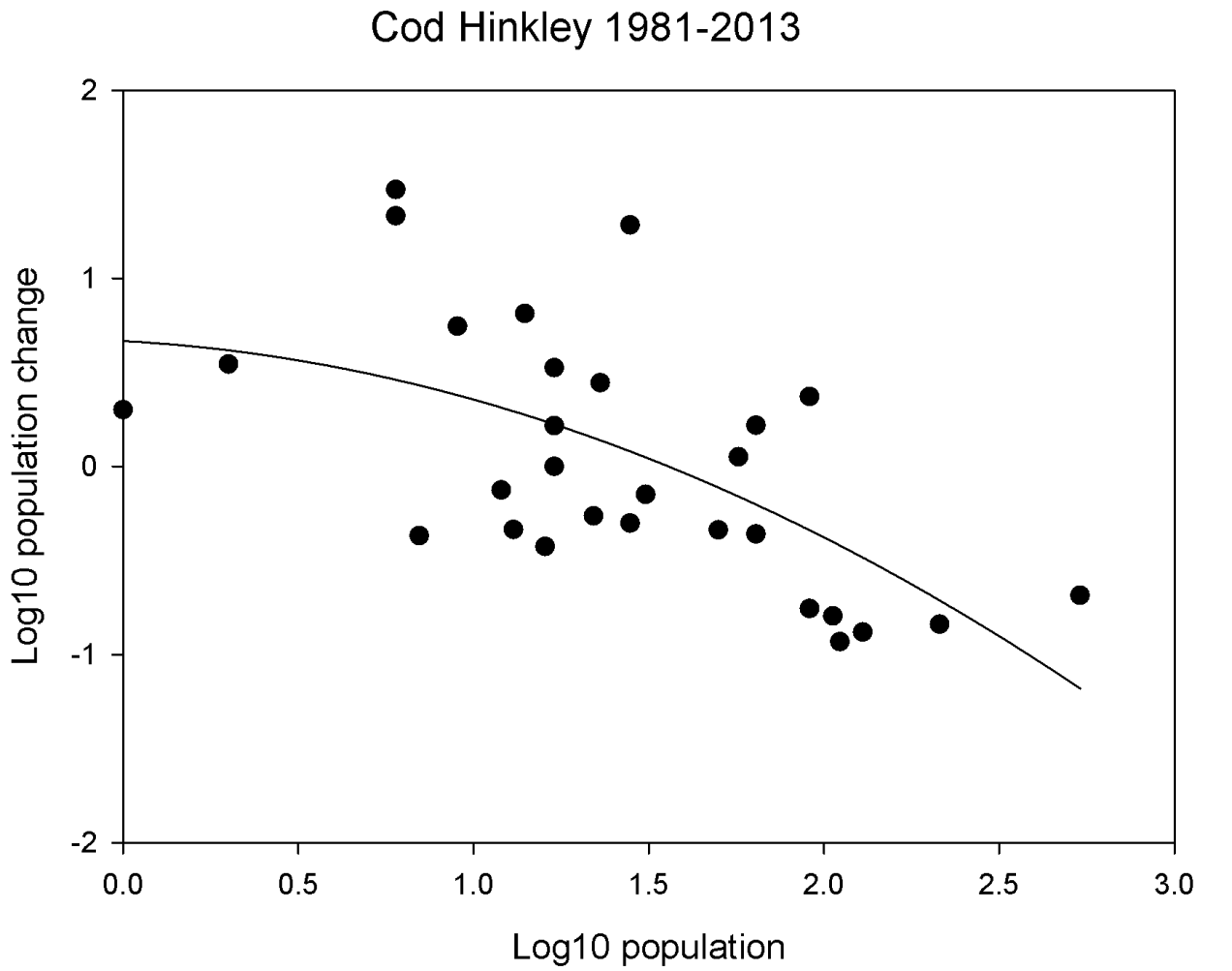


Figure 2 The relationship between population size and change for the cod *Gadus morhua* at Hinkley Point. A quadratic curve was fitted by regression using a best fit algorithm.

### Bulmer’s test

Using count data from 1987 to 2013 inclusive the results for Bulmer’s tests were as follows:

R = 0.53, R_0.5_ = 1.165, **significant for density dependence at 5% level.**

R^*^= -0.31, R_0.5_ = -0.348, **non-significant for density dependence at 5% level.**

Using biomass data from 1992 to 2013 inclusive the results for Bulmer’s tests were as follows:

R = 0.85, R_0.5_ = 0.982, **significant for density dependence at 5% level.**

R^*^= -0.21, R_0.5_ = -0.393, **non-significant for density dependence at 5% level.**

### Additional evidence

As shown in Figure 3 below the total biomass of cod sampled has shown little variation between 1992 and 2012 and shows no significant trend.


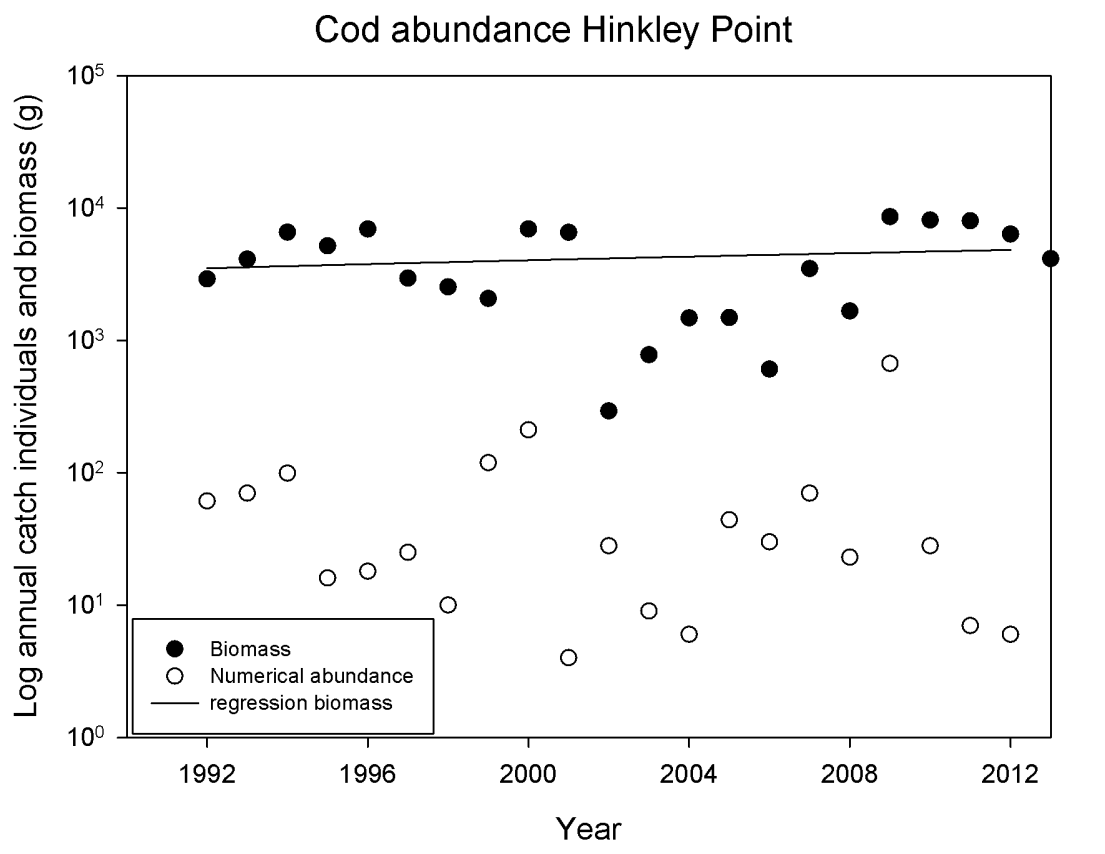


Figure 3 The abundance of cod (Hinkley Point) in count and biomass terms between 1992 and 2012.

## Conger eel, *Conger conger*

Conger eel are notable as they are a top predator and only ever occur in small numbers. The population comprises a considerable number of age classes.

### Relationship between log population change and log population size

The slope of log change in population size offers no proof for density-dependence. While the slope is negative, the gradient of -0.65 is less than -1.0. There is also no evidence of a non-linear relationship or a break-point indicating a threshold response.

**The relationship between Log Population Change and Log Population size gives no support for the existence of density-dependence.**


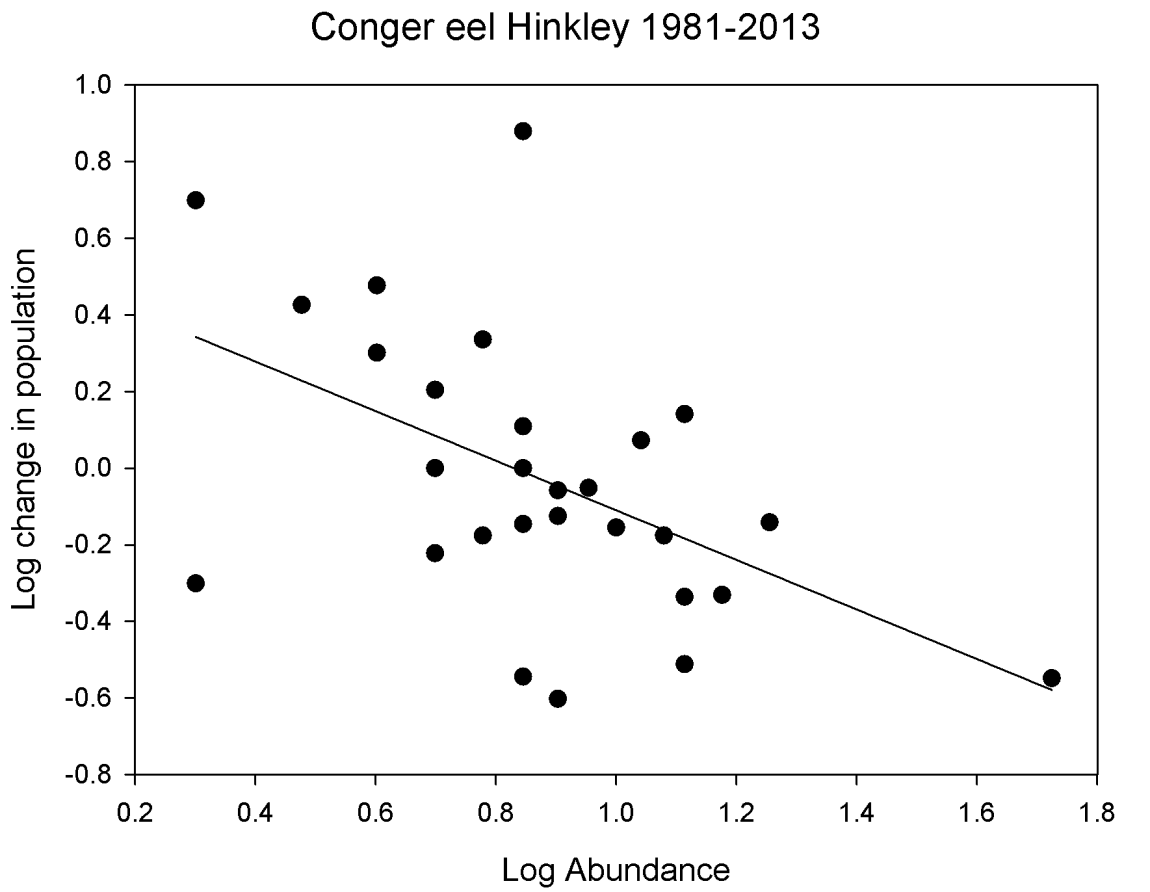


Figure 4 The relationship between population size and change for the conger eel, *Conger conger*, at Hinkley Point. The line was fitted by linear regression.

### Bulmer’s test

Using count data from 1981 to 2013 inclusive the results for Bulmer’s tests were as follows:

R = 0.82, R_0.5_ = 1.38, **significant for density dependence at 5% level.**

R^*^= -0.135, R_0.5_ = -0.304, **non- significant for density dependence at 5% level.**

**Biomass data was not used as biomass of individuals varied greatly from <30 g to > 3000 g**

### Additional evidence

Conger eel are notable as having been caught every year since 1981 in small numbers. The data shows high stability with the exception of a notable peak in recruitment in 2002 (Figure 5). The 53 individuals recorded in 2002 were almost all small, recent, recruits.


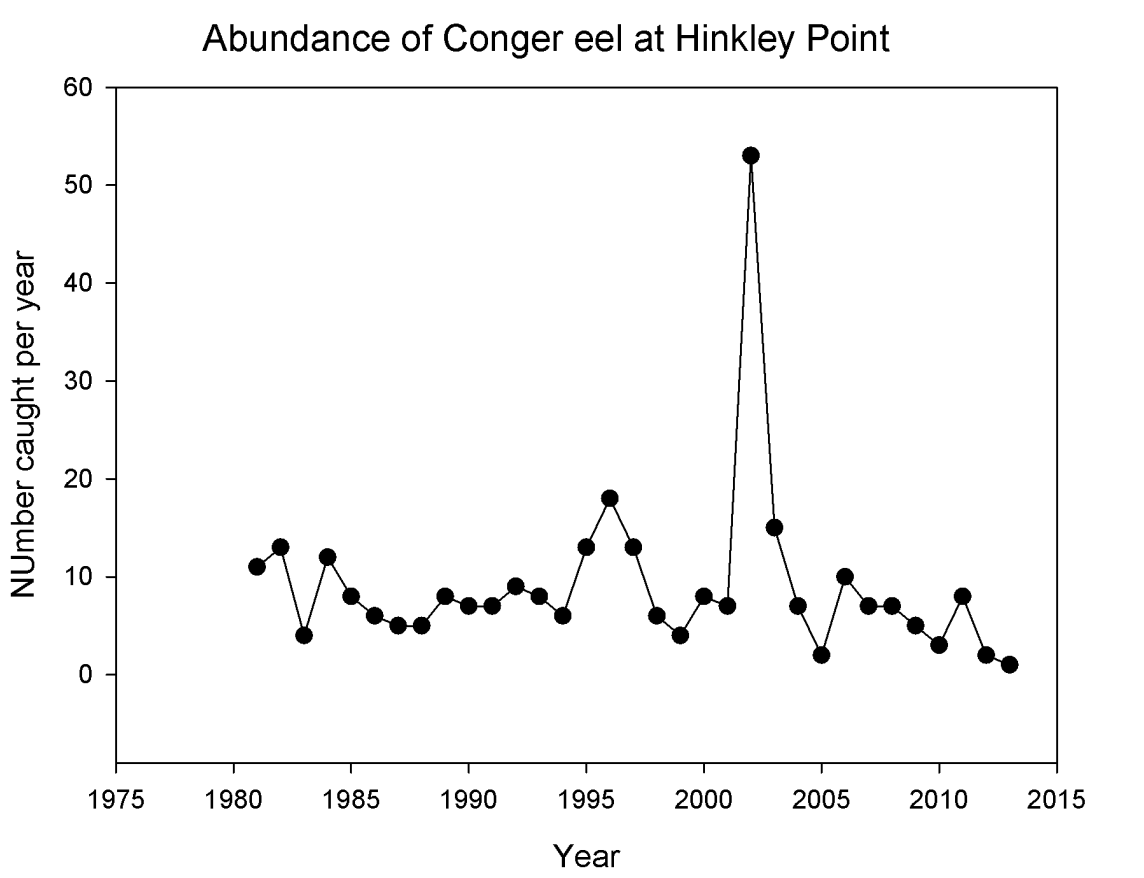


Figure 5 The temporal pattern in Conger eel abundance at Hinkley Point

## Dab, *Limanda limanda*

Dab occur seasonally in the autumn when recently settled fish only a few months old enter the bay for 3-4 months. There has been a distinct discontinuity in dynamics linked to climate change. The species was commoner in the 1980s than in more recent years because it favours cool waters.

### Relationship between log population change and log population size

The slope of log change in population size offers no proof for density-dependence. The log change in populations log population plot shows a negative slope of -0.94 which is insufficiently steep to prove density-dependence, but it is consistent with a density dependent model.

**The relationship between Log Population Change and Log Population size gives no support for the existence of density-dependence.**

Dab are known to be sensitive to water temperature and towards the southern edge of their range in the study area. Climate change with warmer autumn water temperatures after about 1995 produced two distinct periods of abundance at Hinkley Point (Figure 6).


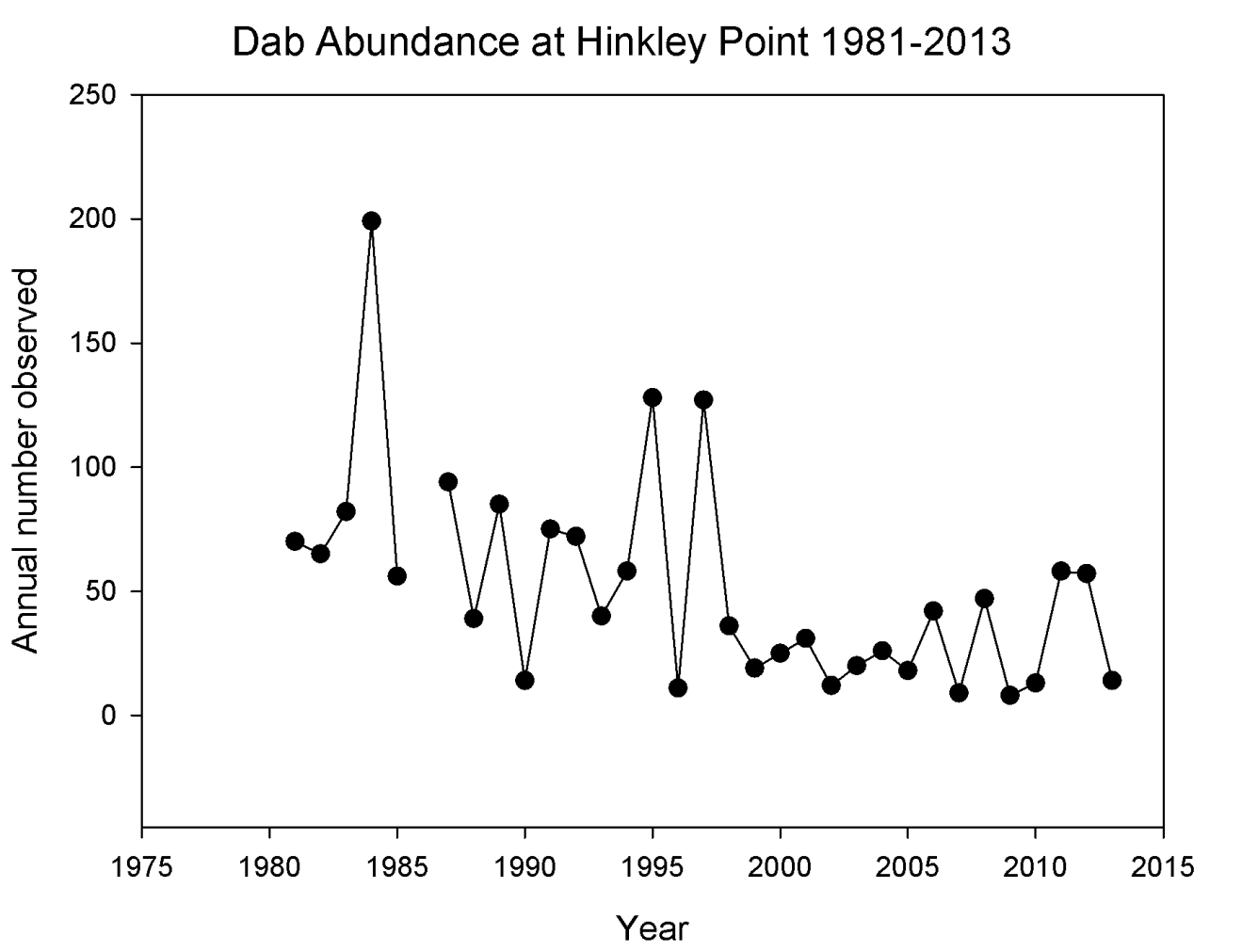


Figure 6 The temporal pattern of abundance of dab at Hinkley Point 1981-2013

However, the major change in abundance at about 1995 indicated that the data should be divided into two sections when this was undertaken (Figure 7) both pre and post 1995 time series gave slopes significantly greater than -1; 1981-1994 has a slope of -1.2 and 1995-2013 has a slope of -1.3.

**There is therefore evidence of density-dependence acting at different levels before and after about 1995.**


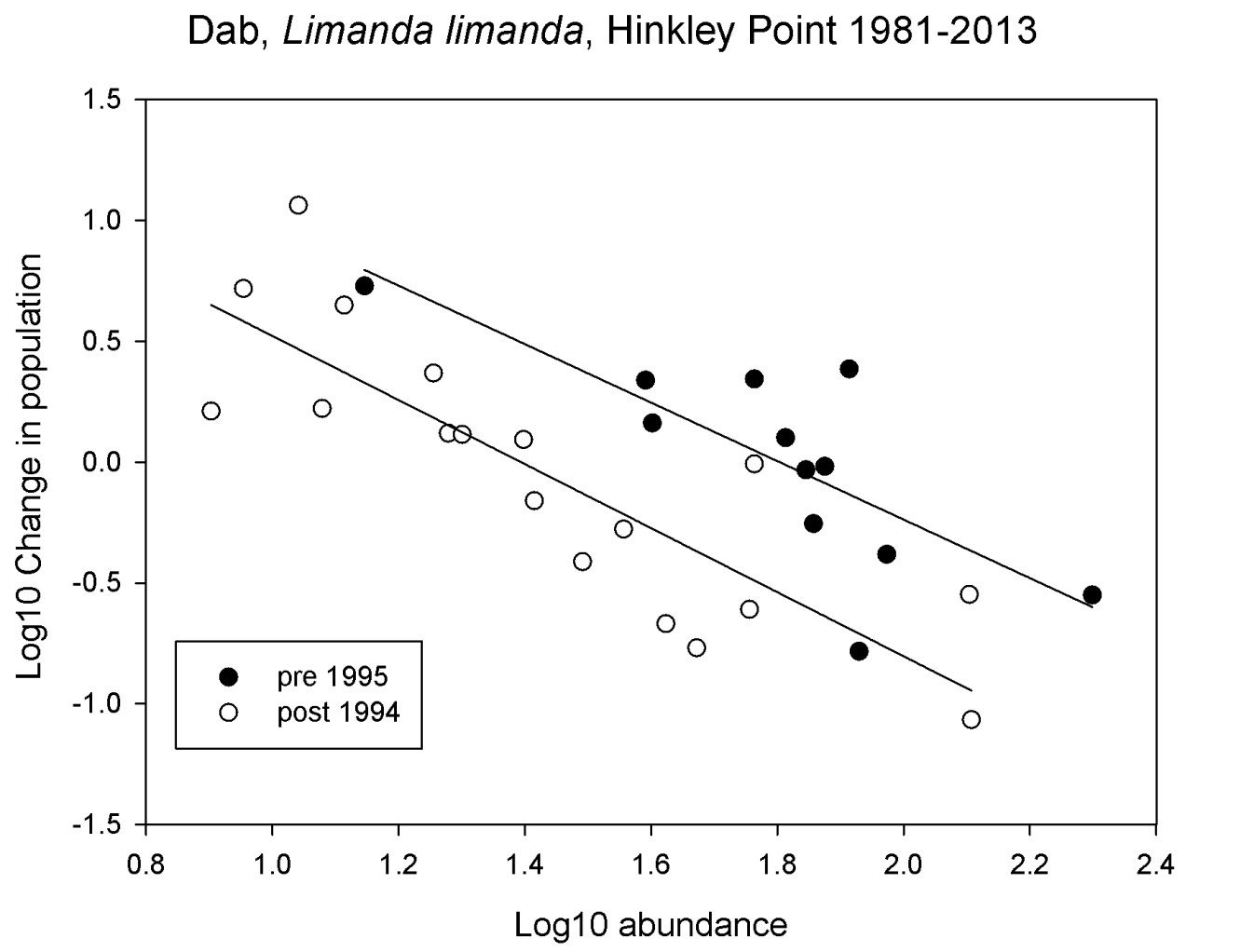


Figure 7 The relationship between population size and change for dab, Limanda limanda, at Hinkley Point. The data have been divided into two periods, 1981-1994 and 1995-2013.

### Bulmer’s test

Using data from 1987 to 2013 inclusive the results for Bulmer’s tests were as follows:

R = 0.46, R_0.5_ = 1.165, **significant for density dependence at 5% level.**

R^*^= 1.165, R_0.5_ = -0.348, **not significant for density dependence at 5% level.**

## Dogfish *Scyliorhinus caniculus*

Dogfish are large and never recorded in large numbers. However, they have been observed every year since 1999 and so Bulmer’s tests could only be applied to this shortened time series of 15 years.

### Relationship between log population change and log population size

The numbers of dogfish caught per year have ranged between 0 and 6 and between 1999 and 2013 the number has ranged between 1 and 6. Nothing can be inferred from such low numbers other than their notable stability. The slope of log change in population size for the years when non-zero numbers were recorded offers no proof for density-dependence. The log change in populations log population plot shows a negative slope of -1.02 which is insufficiently steep to prove density dependence, but it is consistent with a density dependent model.

**The relationship between Log Population Change and Log Population size gives no support for the existence of density-dependence.**

### Bulmer’s test

Using data from 1999 to 2013 inclusive the results for Bulmer’s tests were as follows:

R = 0.57, R_0.5_ = 0.72, **significant for density dependence at 5% level.**

R^*^= -0.02, R_0.5_ = -0.477, **not significant for density dependence at 5% level.**

## Five-Bearded Rocking, *Ciliata mustella*

This is the commonest rockling in Bridgwater Bay and is seasonal in occurrence. The new recruits arrive in the autumn and overwinter in the region.

### Relationship between log population change and log population size

The slope of log change in population size (Figure 8) offers no proof for density-dependence. The log change in populations log population plot shows a negative slope of -0.58 which is insufficiently steep to prove DD. But it is consistent with a density dependent model.

There is also no evidence of a non-linear relationship or a break-point indicating a threshold response.

**The relationship between Log Population Change and Log Population size gives no support for the existence of density-dependence.**


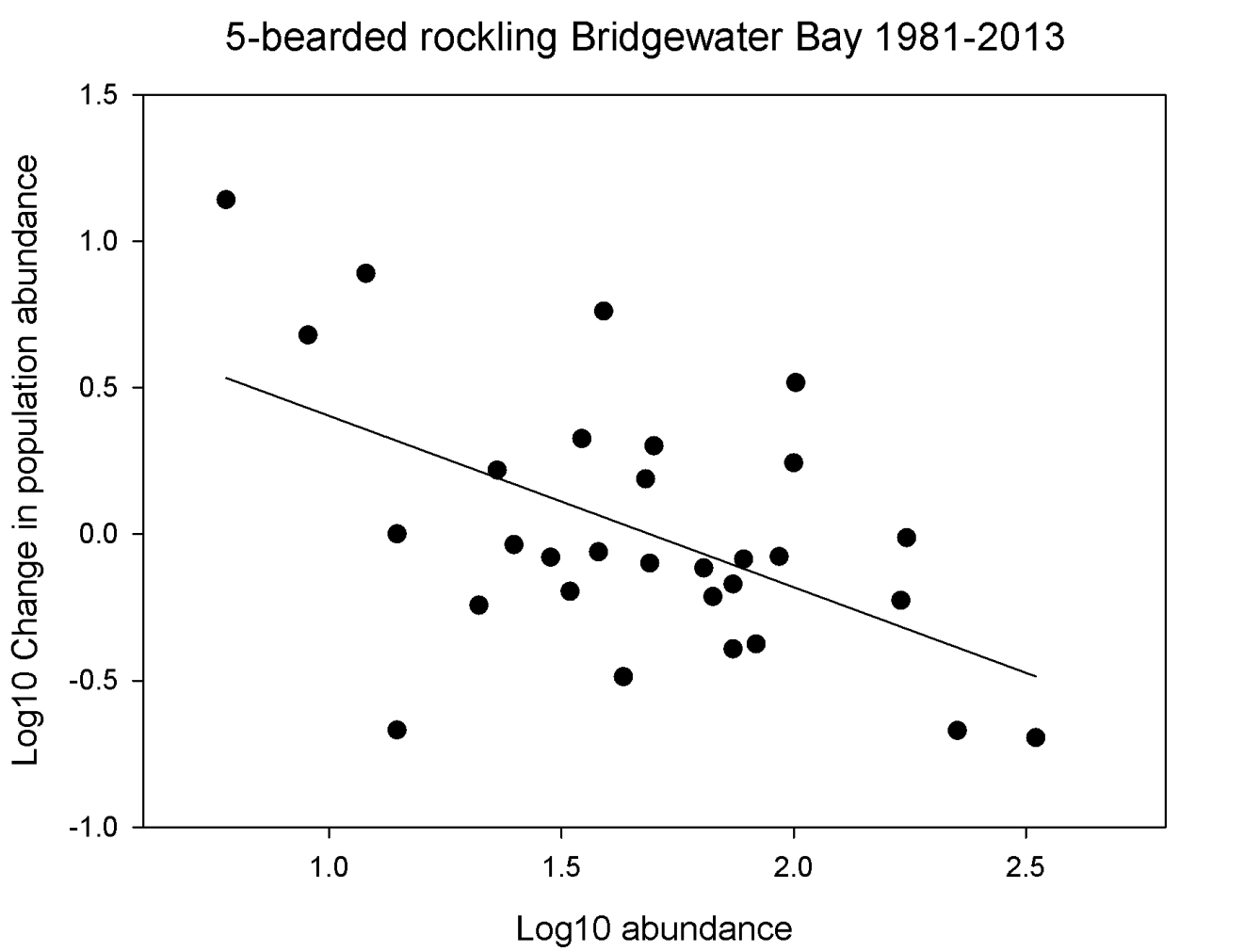


Figure 8 The relationship between population size and change for the 5-bearded rockling *Ciliata mustela* at Hinkley Point.

### Bulmer’s test

Using data from 1987 to 2013 inclusive the results for Bulmer’s tests were as follows:

R = 0.79, R_0.5_ = 1.165, **significant for density dependence at 5% level.**

R^*^= 0.0433, R_0.5_ = -0.348, **non- significant for density dependence at 5% level.**

### Additional evidence

Young of year recruits are observed at Hinkley Point in September to November. Figure 9 shows the relationship between autumn recruit abundance and late winter abundance over January-March. An asymptotic relationship is present indicating that there is a maximum number that can survive the winter irrespective of recruit abundance. This asymptotic relationship gave a good fit to a negative exponential (R^2^ = 0.37).


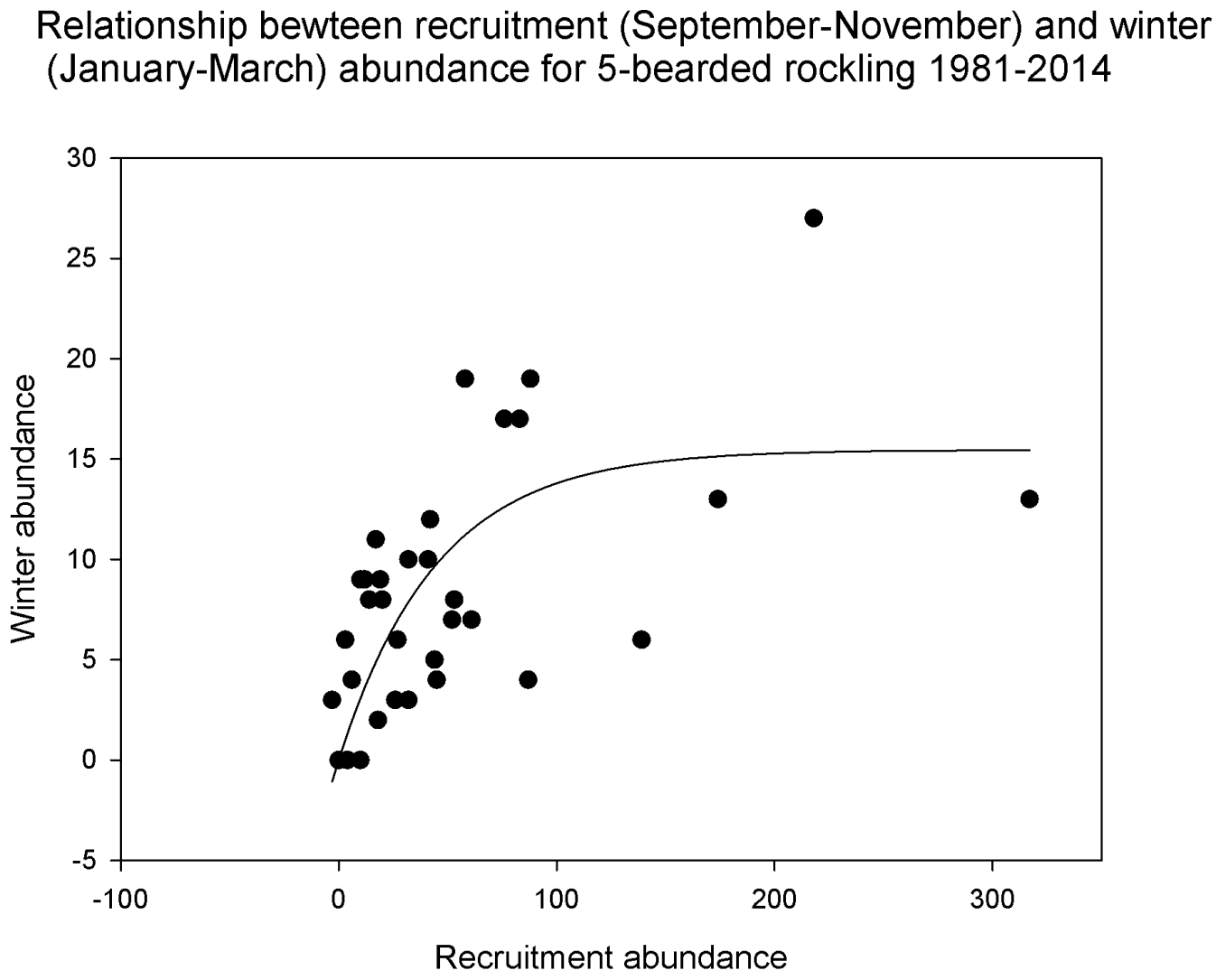


Figure 9 The relationship between recruit abundance and late winter abundance for 5-bearded rockling in Bridgwater Bay 1981-2014.

## Flounder, *Platichthys flesus*

We have the full time series between 1981 and 2014 available for this species as the samples taken in 1986 covered the main period when the species is present in Bridgwater Bay.

### Relationship between log population change and log population size

The relationship between log population change and log population size has a negative slope but does not fit a straight line relationship (Figure 10). The plot shows a fit to a quadratic although there is no biological support for the use of this function.


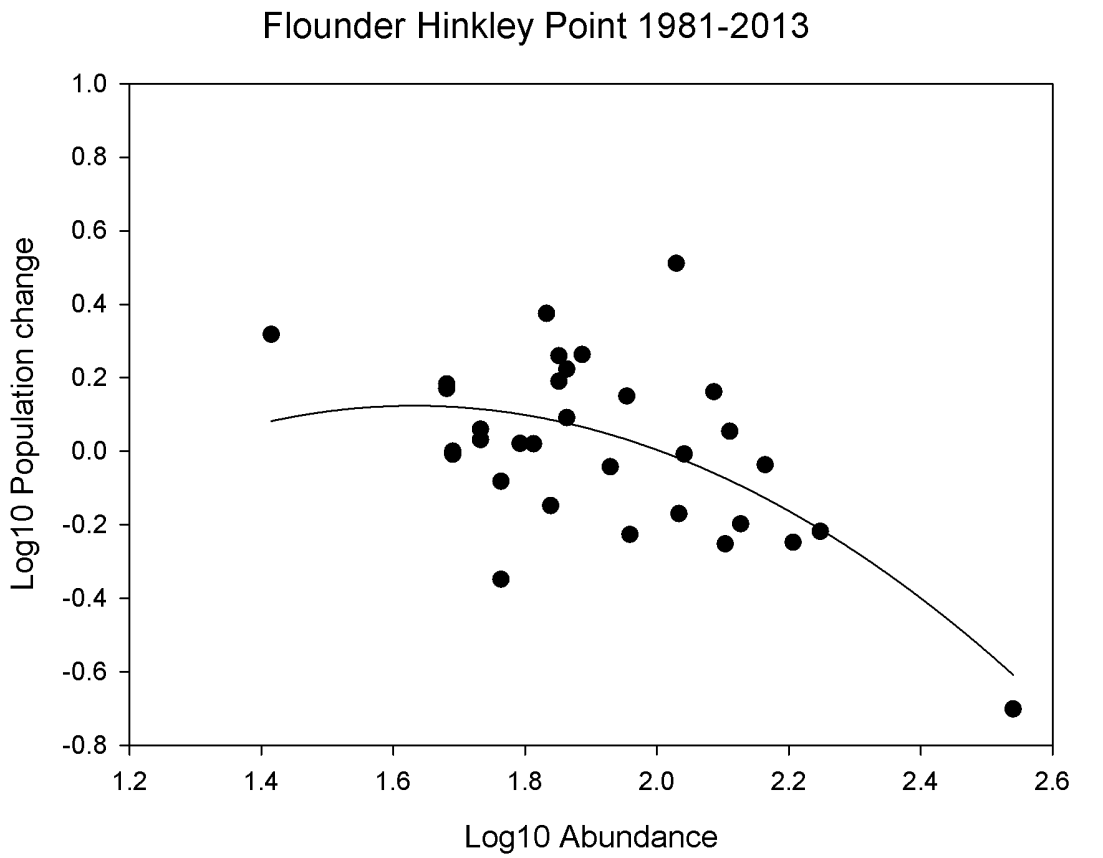


Figure 10 The relationship between population size and change for the flounder *Platichthys flesus* at Hinkley Point. A quadratic curve was fitted by regression using a best fit algorithm.

It is clear that the plot is highly influenced by the single observation at high density and that the scatter in the points will not allow any convincing model to be fitted.

To test for the possible existence of a threshold at about log_10_ abundance of 2.0 the Chow test for structural breaks was undertaken. This gave a test statistic of 3.68 and a probability of 0.038 indicating that the null hypothesis that the two regressions either side of the break are the same should be rejected. Figure 11 shows the fit of a single linear regression and also the two regressions either side of the chosen break point.


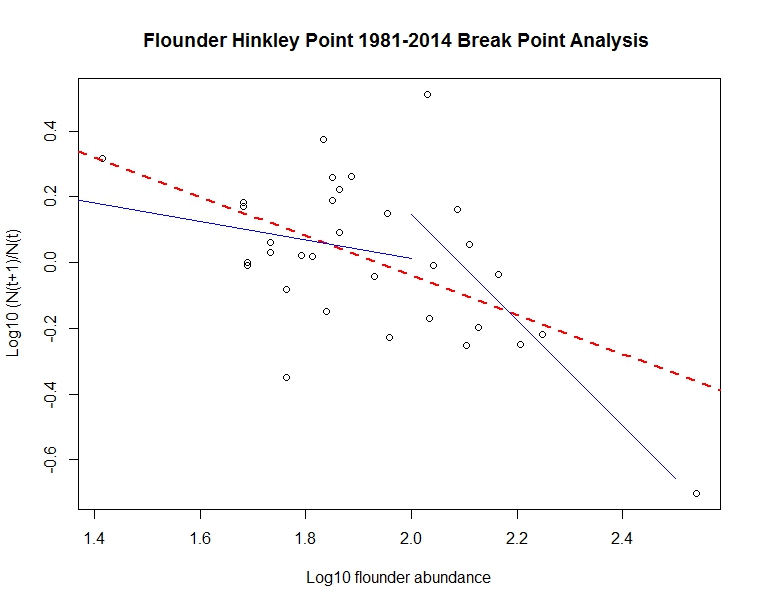


Figure 11 The fitted linear regressions either side of a break point at x=3.3.

**A Non-linear relationship between Log Population Change and Log Population size and a possible break point indicates density-dependence.**

### Bulmer’s test

Using data from 1987 to 2013 inclusive the results for Bulmer’s tests were as follows:

R = 0.85, R_0.5_ = 1.385, **significant for density dependence at 5% level.**

R^*^= -0.26, R_0.5_ = -0.305, **non- significant for density dependence at 5% level.**

## Grey Gurnard *Eutrigla gurnardus*

Grey gurnard are regularly observed in small numbers as juveniles in their first year of life. The dynamics indicate occasional peaks in abundance when recruitment outside Bridgwater Bay is high.


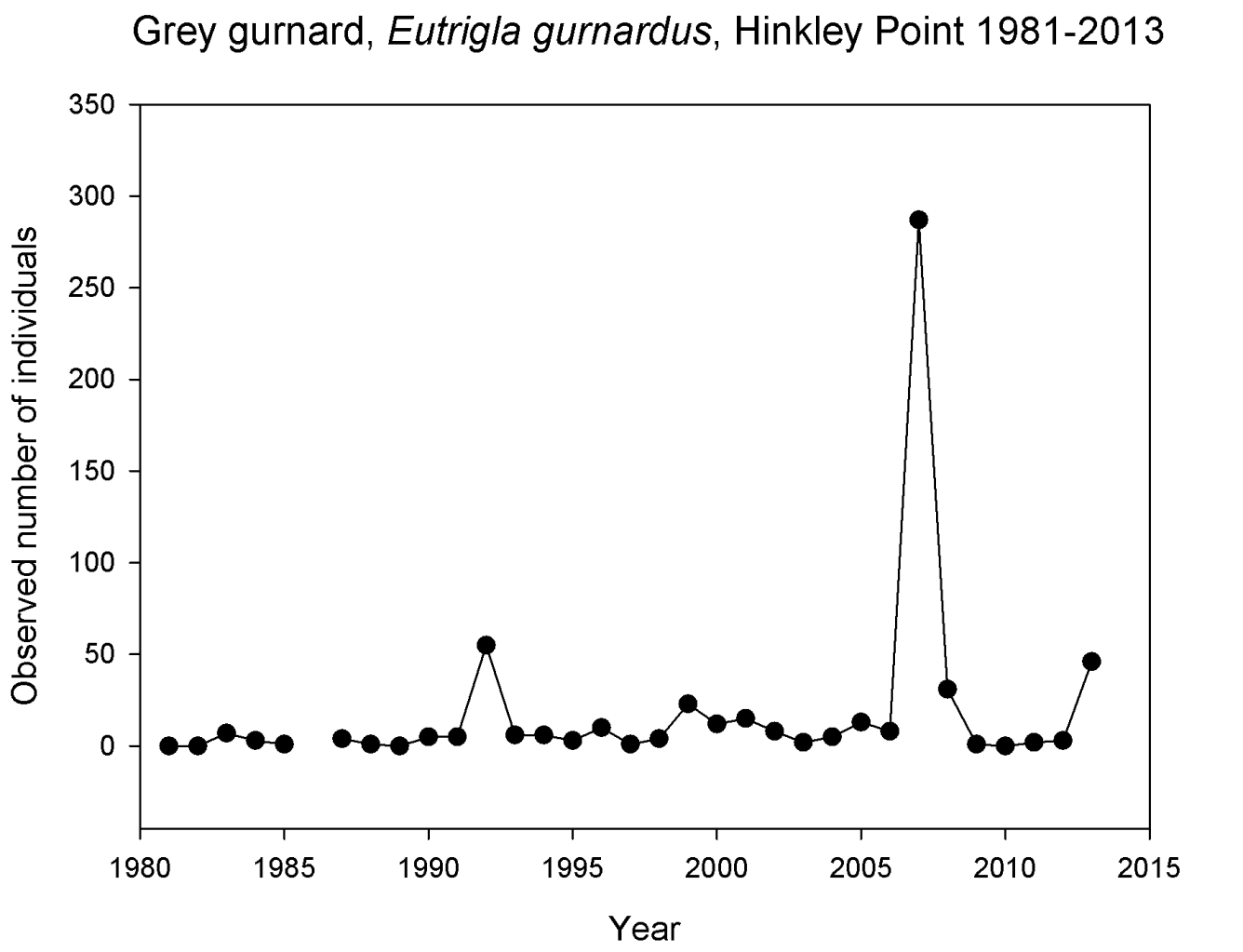


Figure 12 The abundance of grey gurnard, *Eutrigla gurnardus*, at Hinkley Point 1981-2013

### Relationship between log population change and log population size

The slope of log change in population size (Figure 8) offers no proof for density-dependence. The log change in populations log population plot shows a negative slope of -0.78 which is insufficiently steep to prove DD. But it is consistent with a density dependent model.

There is also no evidence of a non-linear relationship or a break-point indicating a threshold response.

**The relationship between Log Population Change and Log Population size gives no support for the existence of density-dependence.**

### Bulmer’s test

Using data from 1987 to 2013 inclusive the results for Bulmer’s tests were as follows:

R = 0.72, R_0.5_ = 1.165, **significant for density dependence at 5% level.**

R^*^= -0.43, R_0.5_ = -0.348, **significant for density dependence at 5% level.**

## Herring *Clupea harengus*

This species occurs primarily as juveniles during the autumn. The species was heavily overfished and by the 1970s the population had collapsed. It is now recovering.

### Relationship between log population change and log population size

The slope of log change in population size offers no proof for density-dependence (Figure 13). The log change in populations log population plot shows a negative slope of -0.653 which is insufficiently steep to prove density-dependence, but it is consistent with a density dependent model.

There is also no evidence of a non-linear relationship or a break-point indicating a threshold response.

**The relationship between Log Population Change and Log Population size gives no support for the existence of density-dependence.**


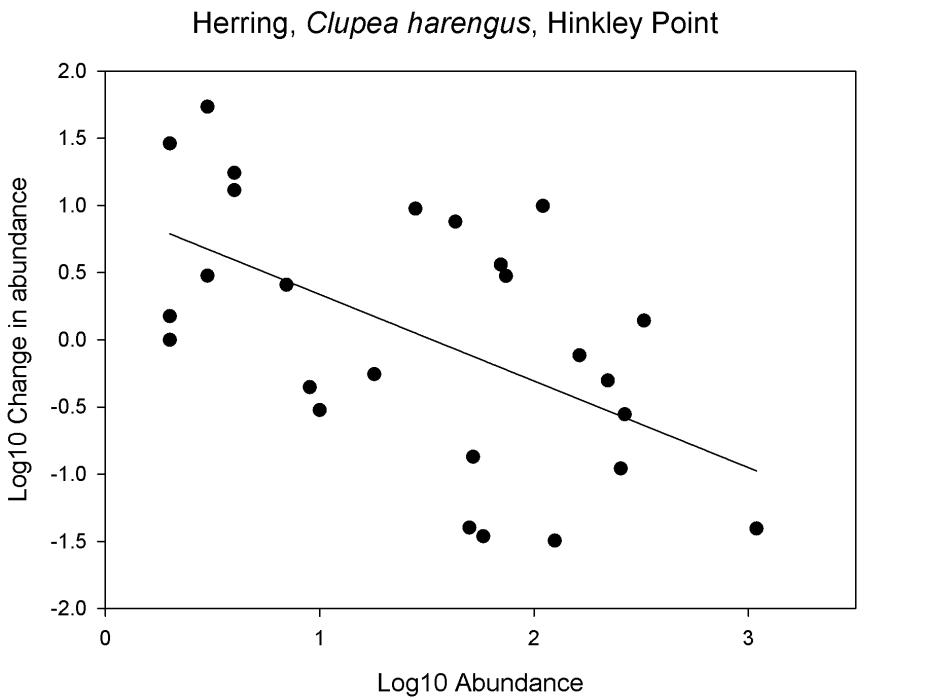


Figure 13 The relationship between population size and change for herring*, Clupea harengus*, at Hinkley Point.

### Bulmer’s test

Using data from 1987 to 2013 inclusive the results for Bulmer’s tests were as follows:

R = 0.79, R_0.5_ = 1.165, **significant for density dependence at 5% level.**

R^*^= 0.1655, R_0.5_ = -0.348, **non**-**significant for density dependence at 5% level.**

### Additional information

The change in abundance shows a recent recovery post 2000 from overfishing (Figure 14).


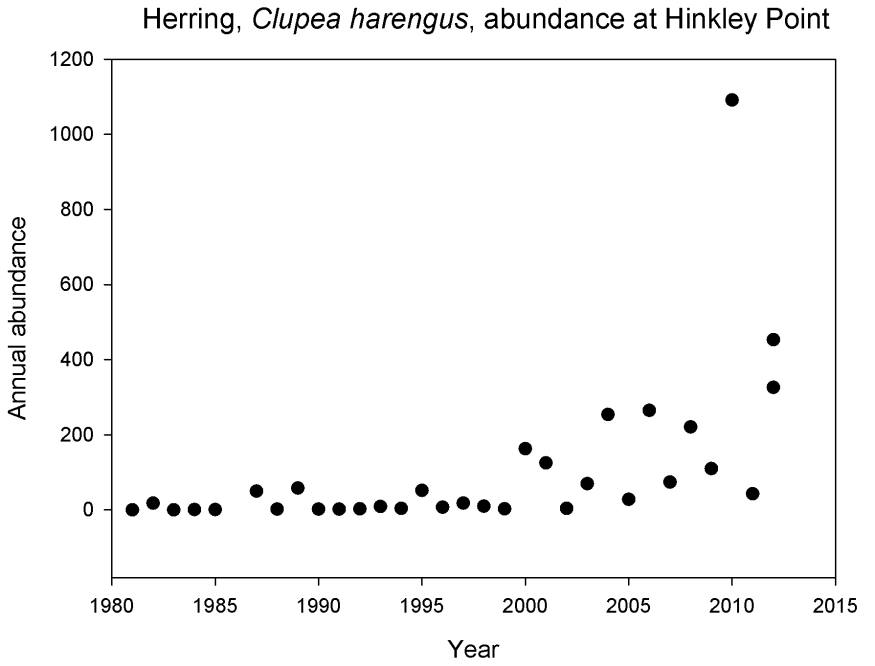


Figure 14 The change in abundance of herring, *Clupea harengus*, at Hinkley Point.

## Hooknose *Agonus cataphractus*

This species occurs in small numbers, but is remarkably stable. Annual numbers have ranged between 1 and 6 between 1981 and 2013 and there have been no zero counts.

### Relationship between log population change and log population size

The slope of log change in population size offers no proof for density-dependence (Figure 15). The log change in populations log population plot shows a negative slope of -0.653 which is insufficiently steep to prove density-dependence, but it is consistent with a density dependent model.

There is also no evidence of a non-linear relationship or a break-point indicating a threshold response.

**The relationship between Log Population Change and Log Population size gives no support for the existence of density-dependence.**


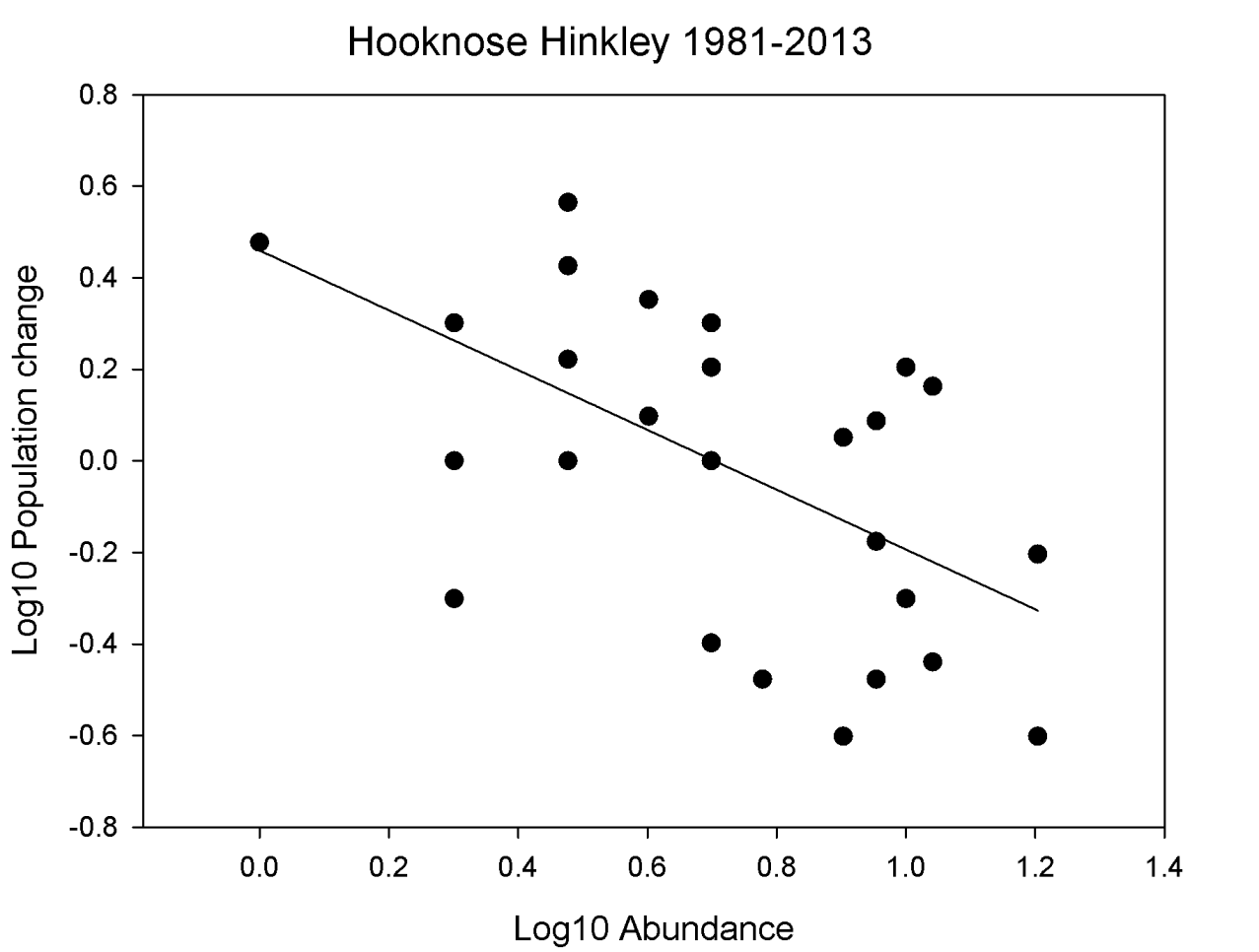


Figure 15 The relationship between population size and change for hooknose*, Agonus cataphractus*, at Hinkley Point.

### Bulmer’s test

Using data from 1987 to 2013 inclusive the results for Bulmer’s tests were as follows:

R = 0.89, R_0.5_ = 1.165, **significant for density dependence at 5% level.**

R^*^= -0.54, R_0.5_ = -0.348, **significant for density dependence at 5% level.**

## Lumpsucker *Cyclopterus lumpus*

This is a cold water species which has declined in abundance since the 1980s. Only 2 individuals have been observed since 2005; it clearly no longer favours Bridgwater Bay.

**Density-dependent control highly unlikely and impossible to assess.**

## Norway Pout *Trisopterus esmarkii*

Norway pout in Bridgwater Bay is a commonly observed low abundance gadoid. It is quite likely that in some years it does not enter the bay as no individuals were caught in 1980, 2010 and 2011. Bulmer’s tests were therefore undertaken on the reduced time series 1987-2009 inclusive.

### Relationship between log population change and log population size

The slope of log change in population size offers no proof for density-dependence (Figure 15). The log change in populations log population plot shows a negative slope of -0.74 which is insufficiently steep to prove density-dependence, but it is consistent with a density dependent model.

There is also no evidence of a non-linear relationship or a break-point indicating a threshold response.

**The relationship between Log Population Change and Log Population size gives no support for the existence of density-dependence.**


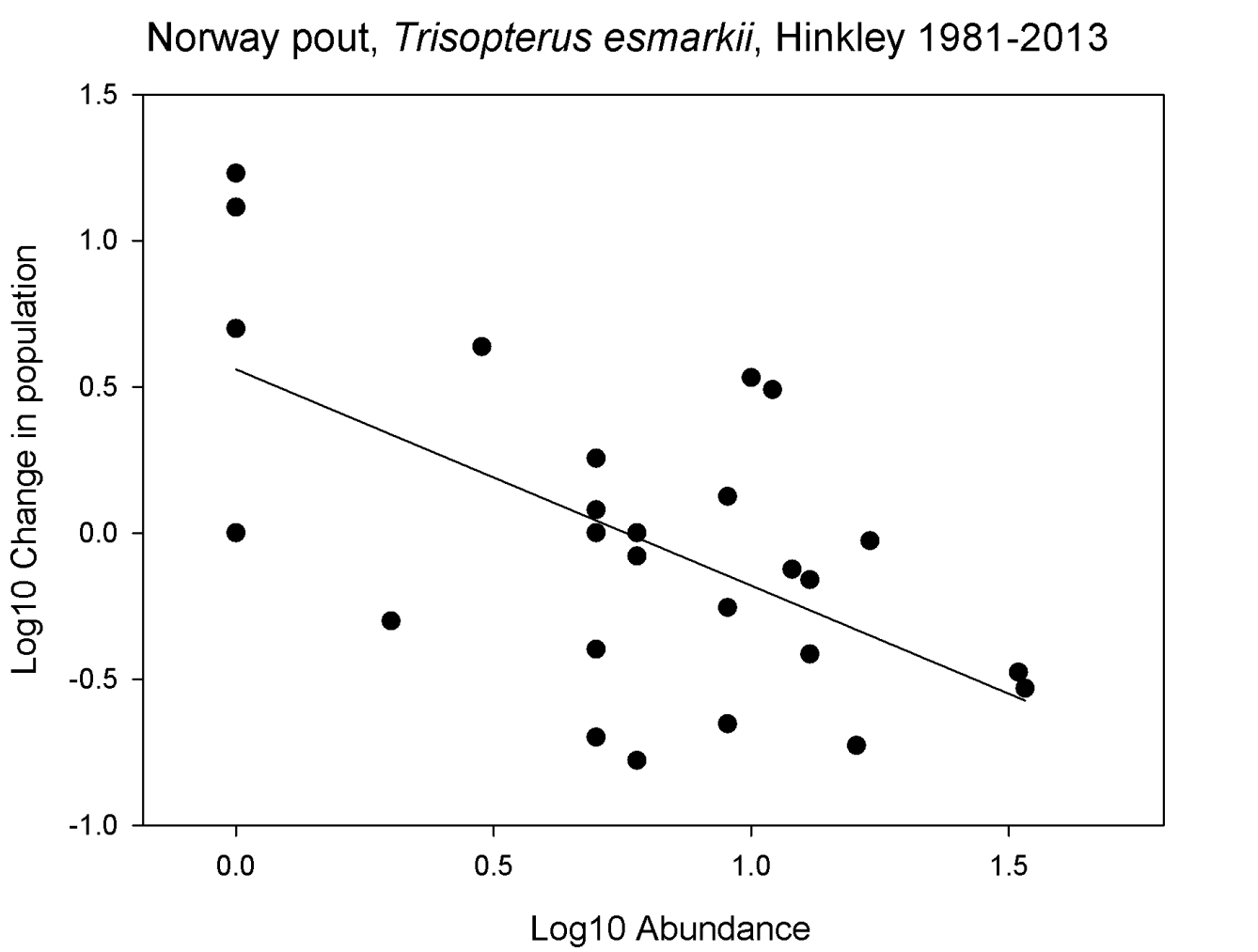


Figure 16 The relationship between population size and change for Norway pout*, Trisopterus esmarkii*, at Hinkley Point.

### Bulmer’s test

Using count data from 1987 to 2013 inclusive the results for Bulmer’s tests were as follows:

R = 0.56, R_0.5_ = 1.101, **significant for density dependence at 5% level**.

R^*^= -0.22, R_0.5_ = -0.38, **non-significant for density dependence at 5% level.**

## Northern rockling, *Ciliata septentrionalis*

This is considered a rare fish in southern British waters. Numbers in Bridgwater Bay are consistently low, yet it is observed most years during winter. A northern species, it probably avoids warm summer waters.

### Relationship between log population change and log population size

Numbers caught per annum have ranged between 0 and 8 since 1981. This analysis was therefore not possible.

### Bulmer’s test

Using count data from 1991to 2011 inclusive the results for Bulmer’s tests were as follows:

R = 0.47, R_0.5_ = 0.95, **significant for density dependence at 5% level.**

R^*^= -0.212, R_0.5_ = -0.4, **non-significant for density dependence at 5% level.**

## Plaice Pleuronectes platessa

Numbers are low and this is one of the least abundant flatfish observed in Bridgwater Bay.

### Relationship between log population change and log population size

The slope of log change in population size offers evidence for density-dependence (Figure 17). The log change in populations – log abundance plot shows a negative slope of -1.12 which is sufficiently greater than -1 to be indicative of density-dependence.

There is also no evidence of a non-linear relationship or a break-point indicating a threshold response.

**The relationship between Log Population Change and Log Population size gives support for the existence of density-dependence.**


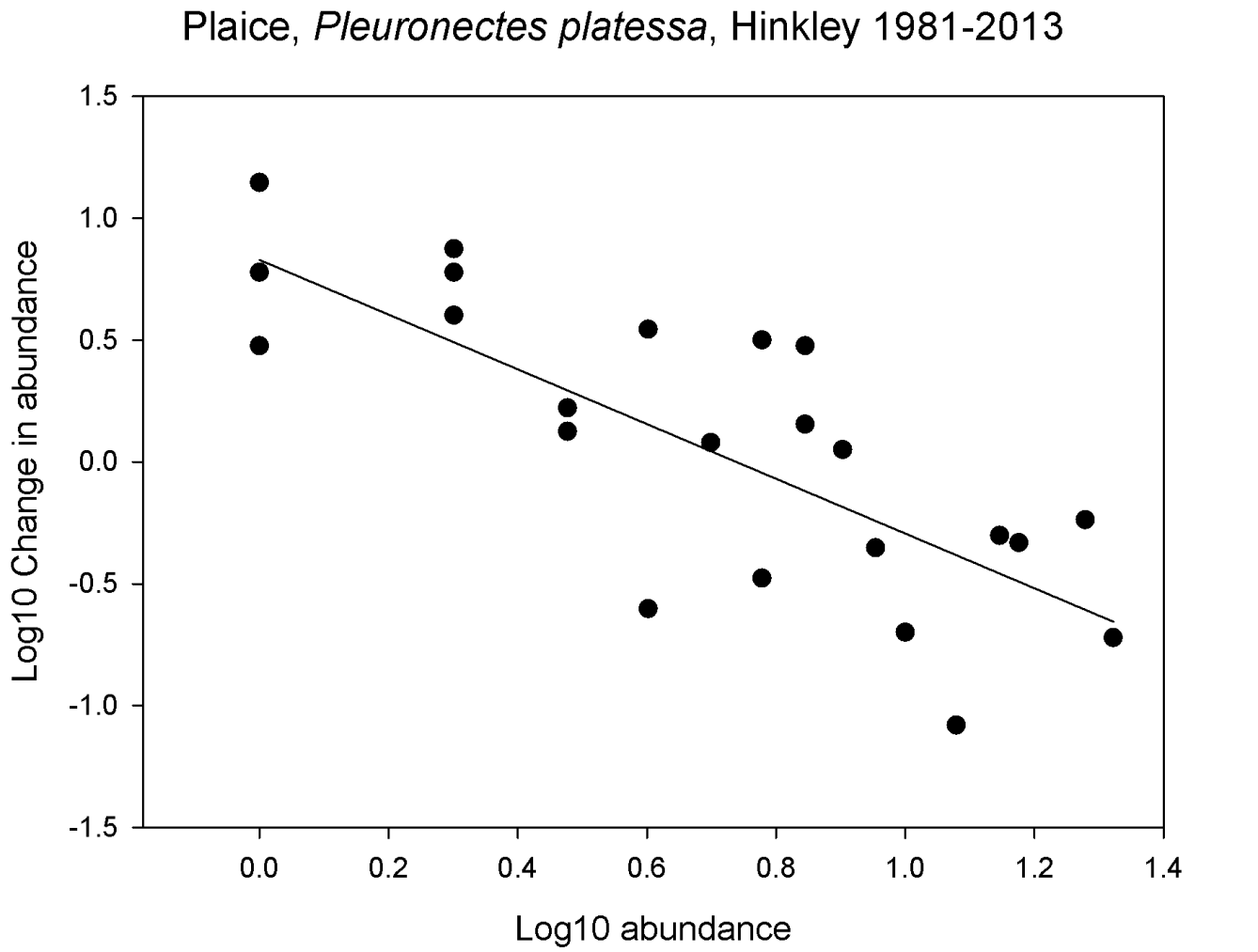


Figure 17 The relationship between population size and change for plaice*, Pleuronectes plattessa*, at Hinkley Point.

### Bulmer’s test

Using count data from 1987 to 2013 inclusive the results for Bulmer’s tests were as follows:

R = 0.47, R_0.5_ = 1.165, **significant for density dependence at 5% level.**

R^*^= 0.055, R_0.5_ = -0.348, **non-significant for density dependence at 5% level.**

## Pollack *Pollachius pollachius*

Pollack is a low abundance, but notably regularly observed gadoid fish. All the individuals caught in Bridgwater Bay are juveniles. Once adult it is likely to be territorial, remaining in a single hunting ground on a reef or wreck.

### Relationship between log population change and log population size

The slope of log change in population size offers evidence for density-dependence (Figure 18). The log change in populations – log abundance plot shows a negative slope of -1.27 which is sufficiently greater than -1 to be indicative of density-dependence.

There is also no evidence of a non-linear relationship or a break-point indicating a threshold response.

**The relationship between Log Population Change and Log Population size gives support for the existence of density-dependence.**


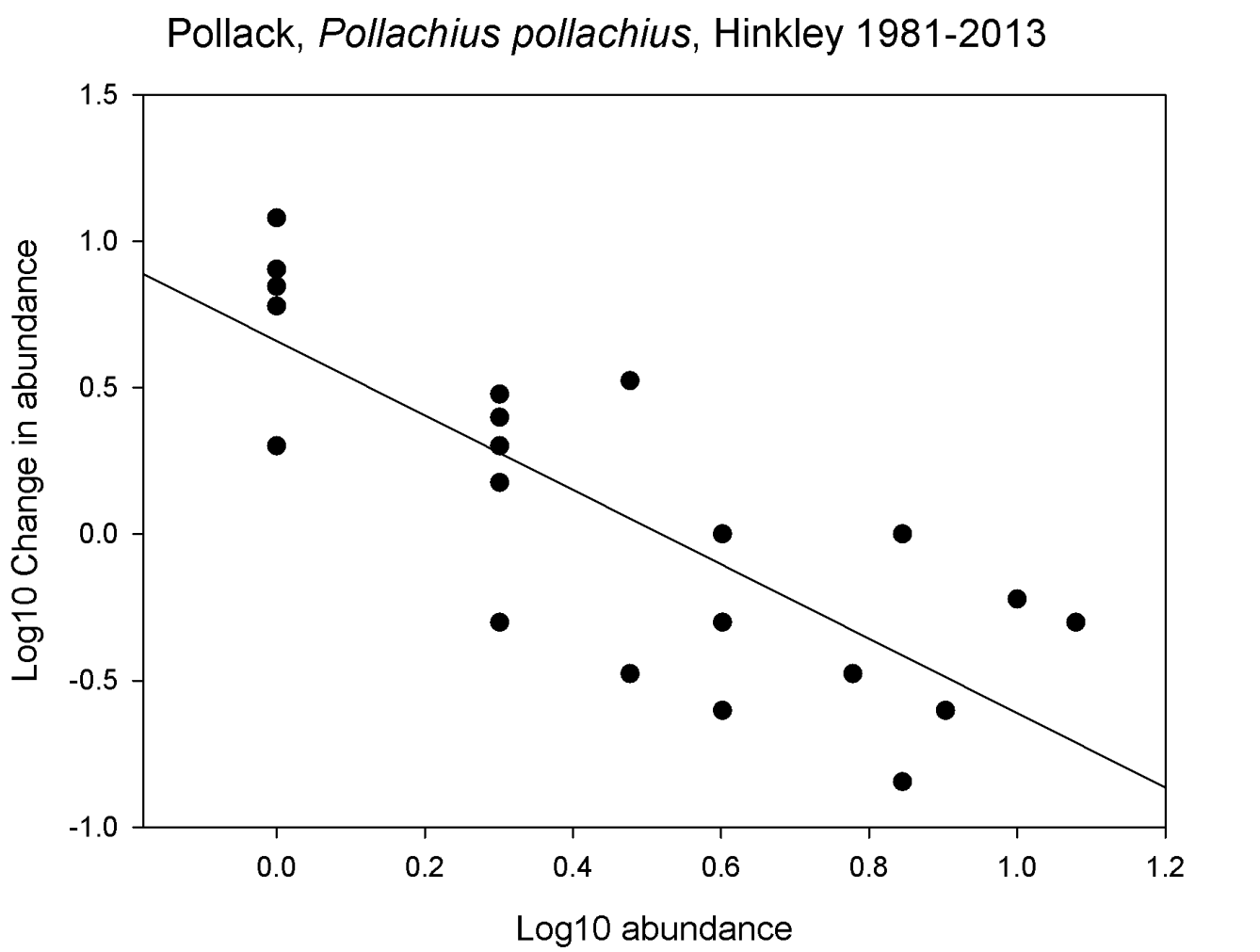


Figure 18 The relationship between population size and change for pollack*, Pollachius pollachius*, at Hinkley Point.

### Bulmer’s test

There are a number of zero observations spaced throughout the time series which make it impossible to reliably apply these tests. The longest run without a zero was 10 years between 1997 and 2006. Bulmer’s test was applied to this reduced series.

R = 0.36, R_0.5_ = 0.54, **significant for density dependence at 5% level.**

R^*^= 0.274, R_0.5_ = -0.593, **non-significant for density dependence at 5% level.**

## Poor cod *Trisopterus minutus*

A highly variable species. The population predominately comprises O-group recruits and recruitment varies between years.

### Relationship between log population change and log population size

The slope of log change in population size (Figure 19) offers no proof for density-dependence. The log change in populations log population plot shows a negative slope of -0.963 which is insufficiently steep to prove density-dependence, but it is consistent with a density dependent model.

There is also no evidence of a non-linear relationship or a break-point indicating a threshold response

**The relationship between Log Population Change and Log Population size gives no support for the existence of density-dependence.**


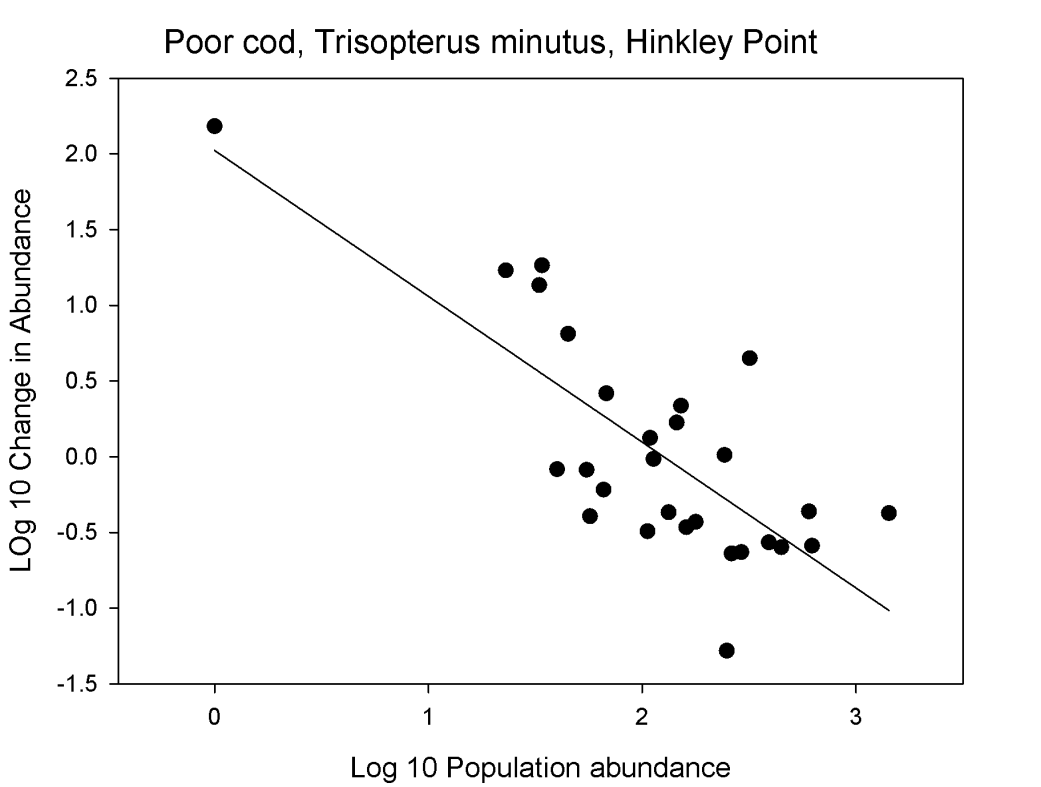


Figure 19 The relationship between population size and change for poor cod.

### Bulmer’s test

Using count data from 1999 to 2013 inclusive the results for Bulmer’s tests were as follows:

R = 0.78, R_0.5_ = 1.165, **significant for density dependence at 5% level.**

R^*^= -0.509, R_0.5_ = -0.348, **significant for density dependence at 5% level.**

### Additional evidence

While showing great between years variability, poor cod have shown no long-term trend in abundance (Figure 20). There is a hint of a long-term decline. However, this seems to be because of recruitment failure in 2009/10/11 which has been followed by a bounce back in 2012.


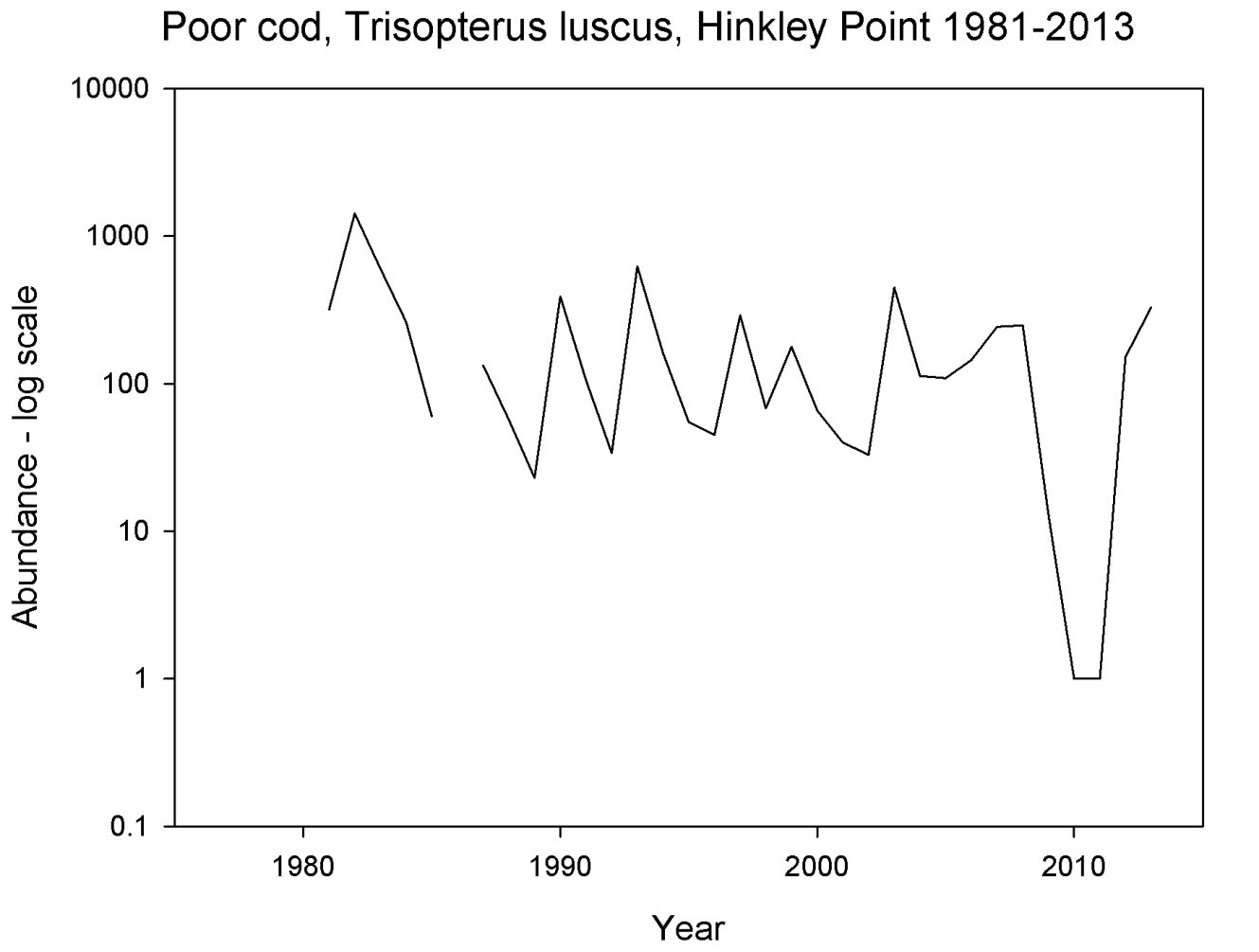


Figure 20 The abundance of poor cod, *Trisopterus minutus*, at Hinkley Point 1981-2013.

## Pout, *Trisopterus luscus*

### Relationship between log population change and log population size

The slope of log change in population size (Figure 21) offers no proof for density-dependence. The log change in populations log population plot shows a negative slope of -0.58 which is insufficiently steep to prove DD. But it is consistent with a density dependent model.

There is also no evidence of a non-linear relationship or a break-point indicating a threshold response.

**The relationship between Log Population Change and Log Population size gives no support for the existence of density-dependence.**


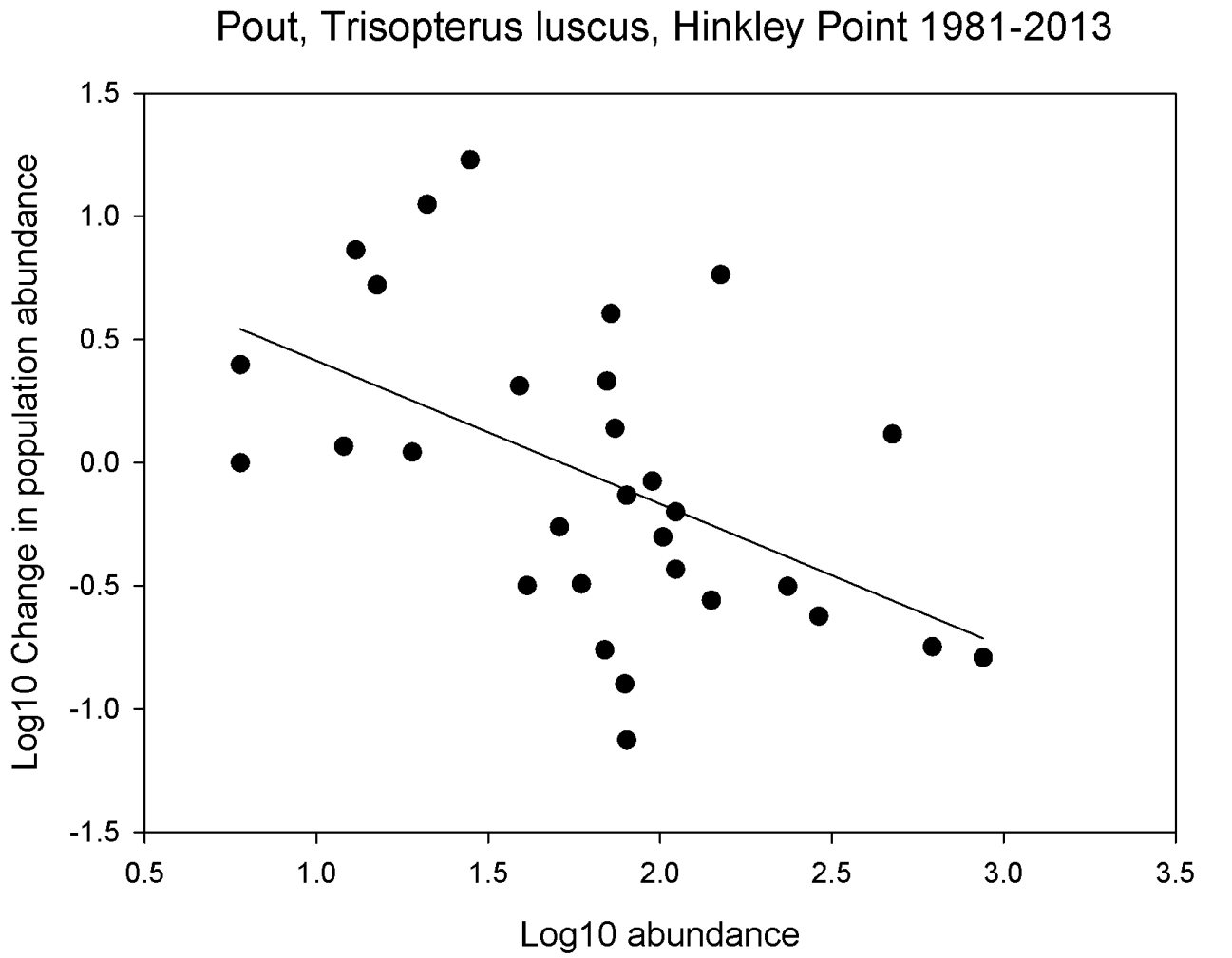


Figure 21 The relationship between population size and change for pout, *Trisopterus luscus*, at Hinkley Point.

### Bulmer’s test

Using count data from 1987 to 2013 inclusive the results for Bulmer’s tests were as follows:

R = 0.85, R_0.5_ = 1.165, **significant for density dependence at 5% level.**

R^*^= -0.405, R_0.5_ = -0.348, **significant for density dependence at 5% level.**

### Additional evidence

Pout abundance varies considerably between years almost certainly related to large changes in recruitment success as almost all the pout observed in Bridgwater Bay are in their first year of life. It is clear that strong recruitment years are followed by weak years and the result is a population which shows no trend (Figure 22). Pout numbers show no long-term trend. The slight decline in the linear regression is not significant.


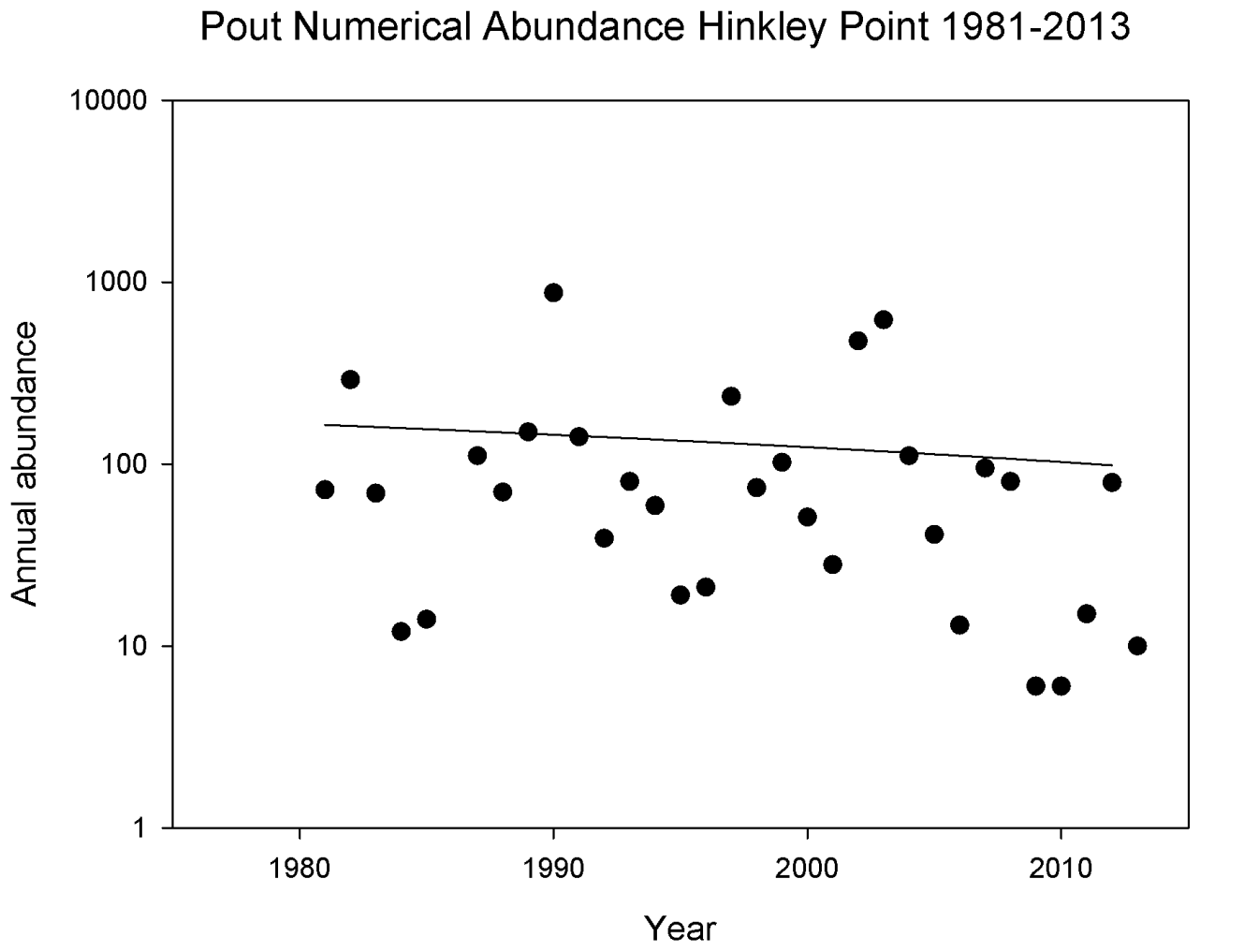


Figure 22 The abundance of pout, *Trisopterus luscus*, at Hinkley Point 1981-2013.

## Sand goby *Pomatoschistus minutus*

This is a common species probably only living for about 15 months. The entire life cycle occurs in Bridgwater Bay.

### Relationship between log population change and log population size

The slope of log change in population size (Figure 23) offers no proof for density-dependence. The log change in populations log population plot shows a negative slope of -0.67 which is insufficiently steep to prove density-dependence, but it is consistent with a density dependent model.

There is also no evidence of a non-linear relationship or a break-point indicating a threshold response.

**The relationship between Log Population Change and Log Population size gives no support for the existence of density-dependence.**


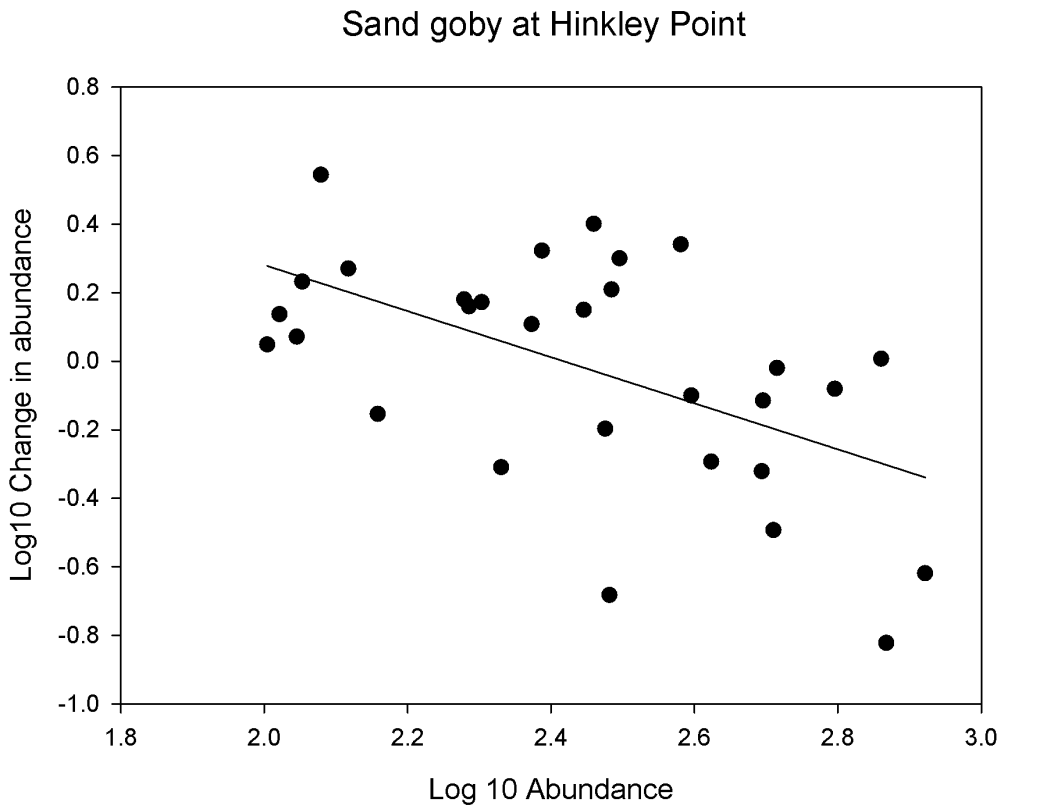


Figure 23 The relationship between population size and population change for sand goby.

### Bulmer’s test

Using count data from 1999 to 2013 inclusive the results for Bulmer’s tests were as follows:

R = 0.87, R_0.5_ = 1.385, **significant for density dependence at 5% level.**

R^*^= -0.317, R_0.5_ = -0.305, **significant for density dependence at 5% level.**

### Additional evidence

While showing considerable between years variability, sand goby have shown no long-term trend in abundance (Figure 20).


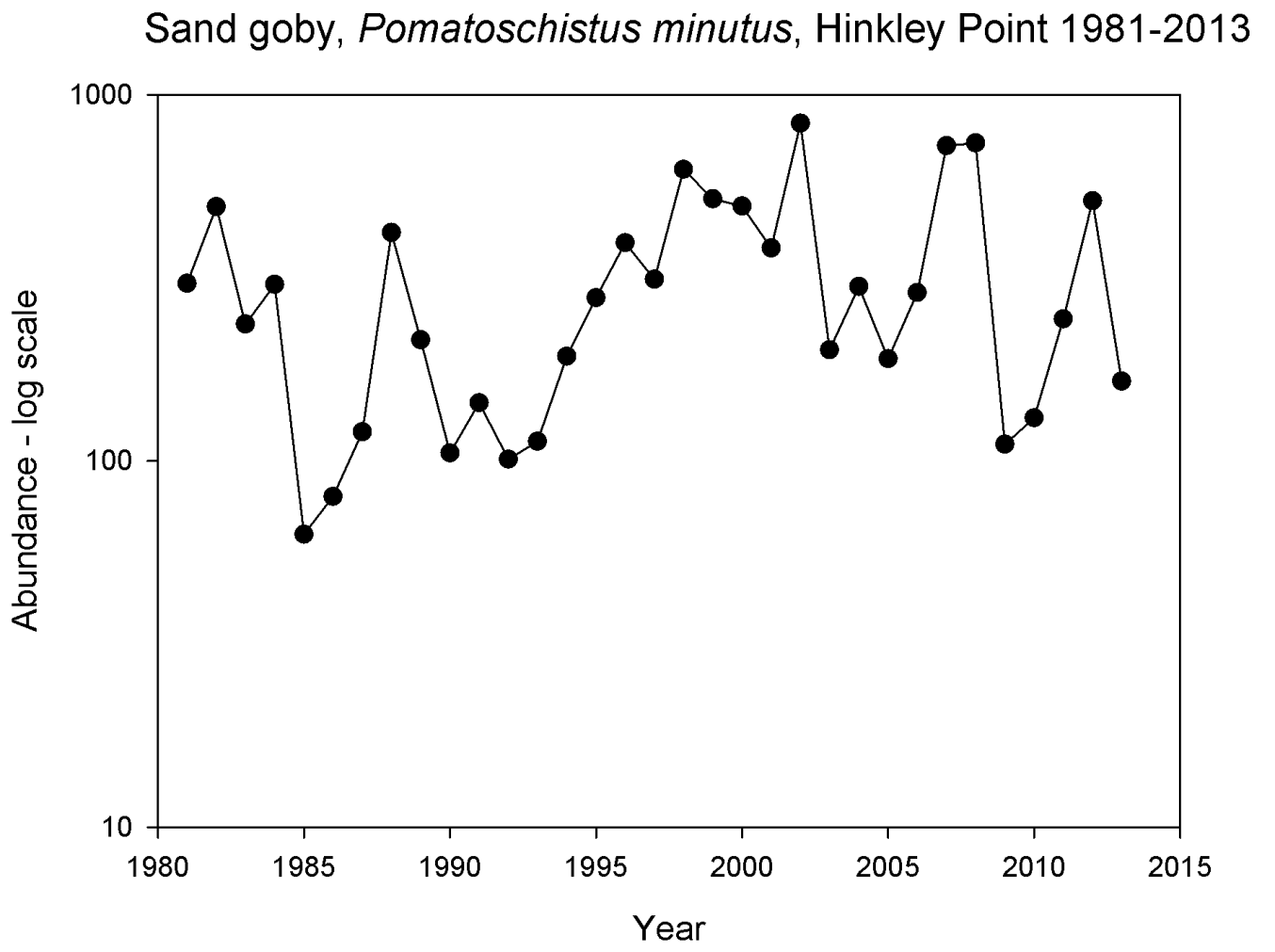


Figure 24 The abundance of sand goby, *Pomatoschistus minutus*, at Hinkley Point 1981-2013.

## Sea snail *Liparis liparis*

### Relationship between log population change and log population size

The slope of log change in population size (Figure 25) offers no proof for density-dependence. The log change in populations log population plot shows a negative slope of -0.54 which is insufficiently steep to prove DD. But it is consistent with a density dependent model.

There is also no evidence of a non-linear relationship or a break-point indicating a threshold response.

**The relationship between Log Population Change and Log Population size gives no support for the existence of density-dependence.**


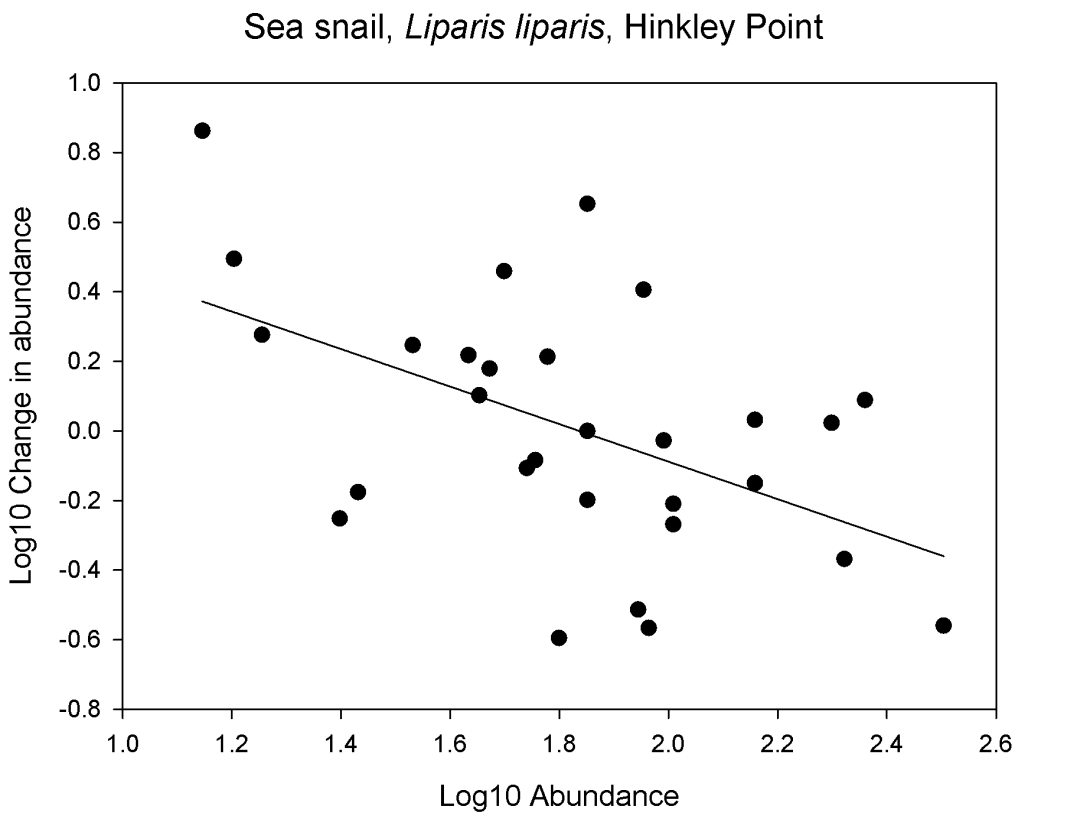


Figure 25 The relationship between population size and change for pout, *Trisopterus luscus*, at Hinkley Point.

### Bulmer’s test

Using count data from 1987 to 2013 inclusive the results for Bulmer’s tests were as follows:

R = 0.77, R_0.5_ = 1.165, **significant for density dependence at 5% level.**

R^*^= -0.64, R_0.5_ = -0.348, **significant for density dependence at 5% level.**

### Additional evidence

The study of Henderson & Seaby [[5](#_ENREF_5)] demonstrated that abundance varied with temperature and there was an underlying remarkable stability.

## Snake pipefish *Entelurus aequoreus*

The dynamics of this species are extraordinary. There is a single extreme peak in abundance with the maximum recorded in 2008 (Figure 26). By 2013 the count had declined to zero. The outburst of pipefish in the East Atlantic and North Sea has been widely reported and the reasons are unknown. It is likely that the explosive increase and decline observed is a function of the concentration of the animals linked to wind and tide. This is an open water pelagic pipefish and therefore the production of a large area of open ocean can be concentrated in coastal waters if tide, current and wind allow.

It is clear that conditions within Bridgwater Bay are not determining abundance and there is therefore no local density-dependent control.


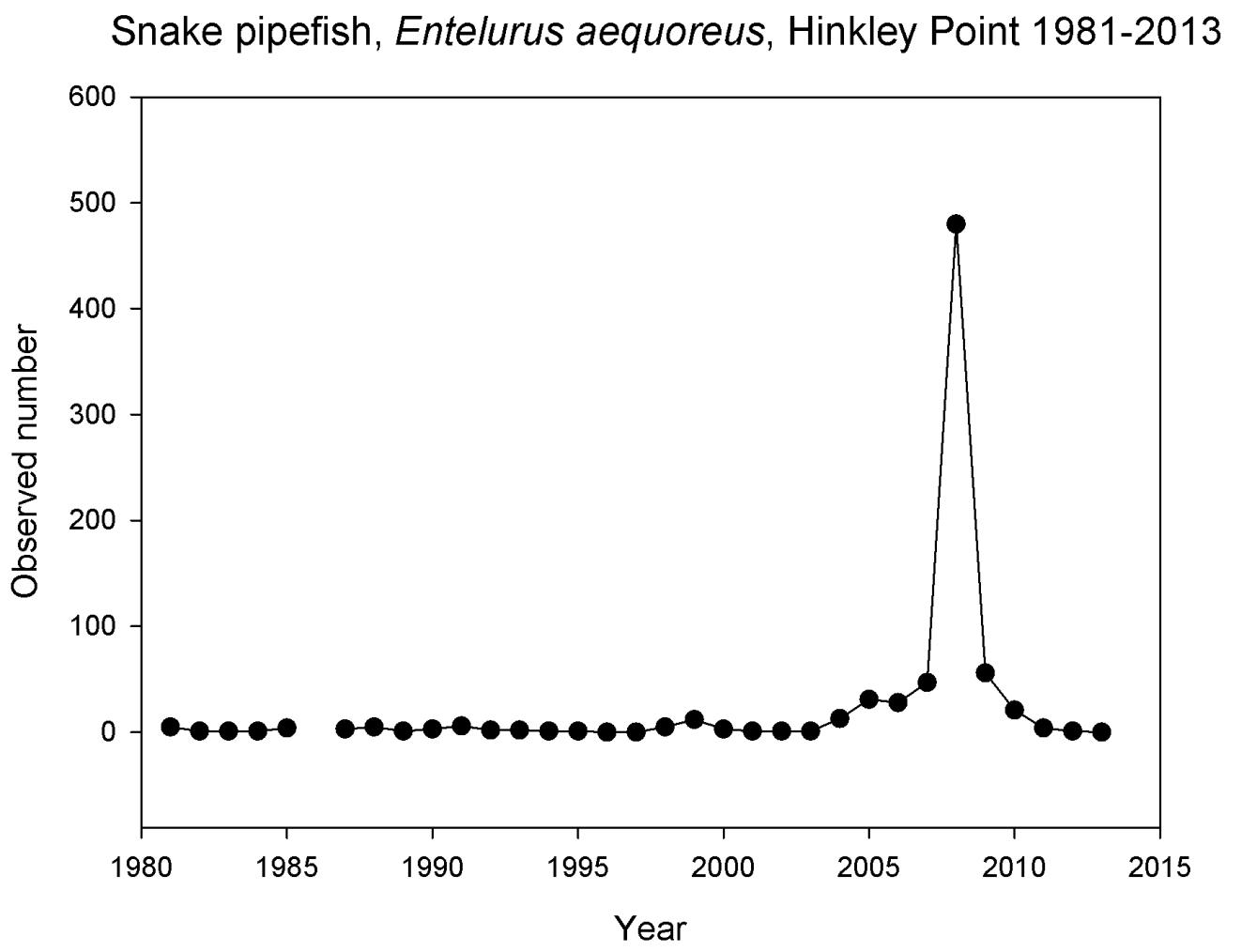


Figure 26 The abundance of snake pipefish, *Entelurus aequoreus*, at Hinkley Point.

## Sole, *Solea solea*

This is one of the most abundant flatfish and are known to have increased in abundance since 1980. This increase is correlated with increased water temperature in the spring.

### Relationship between log population change and log population size

The slope of log change in population size (Figure 27) offers no proof for density-dependence. The log change in populations log population plot shows a negative slope of -0.4 which is insufficiently steep to prove density-dependence, but it is consistent with a density dependent model.

There is also no evidence of a non-linear relationship or a break-point indicating a threshold response.

**The relationship between Log Population Change and Log Population size gives no support for the existence of density-dependence.**


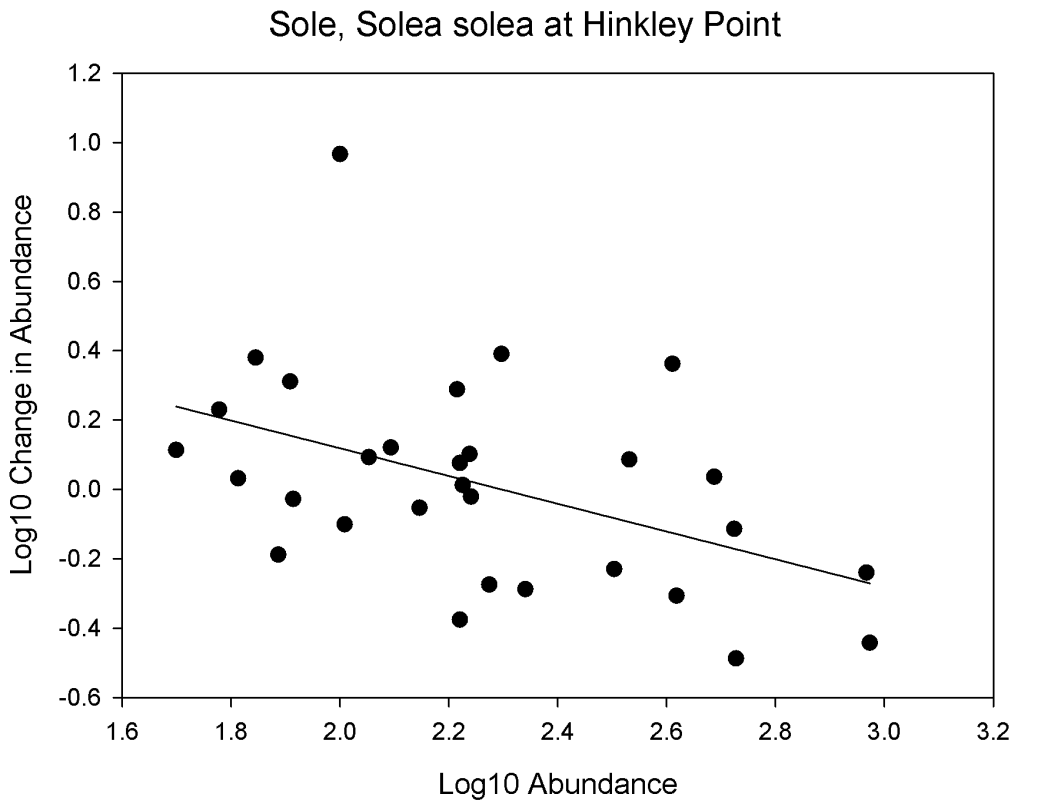


Figure 27 The relationship between population size and population change for sole, Solea solea.

### Bulmer’s test

Using count data from 1987 to 2013 inclusive the results for Bulmer’s tests were as follows:

R = 1.02, R_0.5_ = 1.165, **significant for density dependence at 5% level.**

R^*^= -0.145, R_0.5_ = -0.348, **non-significant for density dependence at 5% level.**

### Additional evidence

Sole first appear in Bridgwater Bay during the summer and a recruitment index is the sum of the number of 0-group individuals recorded in samples collected between July and December. They tend to move offshore during the winter and reappear in April. The sum of the sole collected between April to June gives a measure of the abundance after their first winter.

As Figure 28 below shows, using these two measures of abundance before and after their first winter sole show clear density dependent overwintering survival. The greater the number of recruits during their first summer the greater the overwintering mortality (instantaneous mortality is plotted as a negative value). The linear regression line is significant (see stats below).

**R Rsqr Adj Rsqr Standard Error of Estimate**

0.3979 0.1583 0.1260 0.6505

**Coefficient Std. Error t P**

y0 0.2175 0.6760 0.3218 0.7502

a -0.3058 0.1383 -2.2116 0.0360

**Analysis of Variance:**

Analysis of Variance:

**DF SS MS**

Regression 2 46.0013 23.0007

Residual 26 11.0005 0.4231

Total 28 57.0018 2.0358

Corrected for the mean of the observations:

**DF SS MS F P**

Regression 1 2.0694 2.0694 4.8912 0.0360

Residual 26 11.0005 0.4231

Total 27 13.0699 0.4841

**Statistical Tests:**

**Normality Test (Shapiro-Wilk)** Failed (P = 0.0336)

W Statistic= 0.9194 Significance Level = 0.0500

**Constant Variance Test** Passed (P = 0.1841)


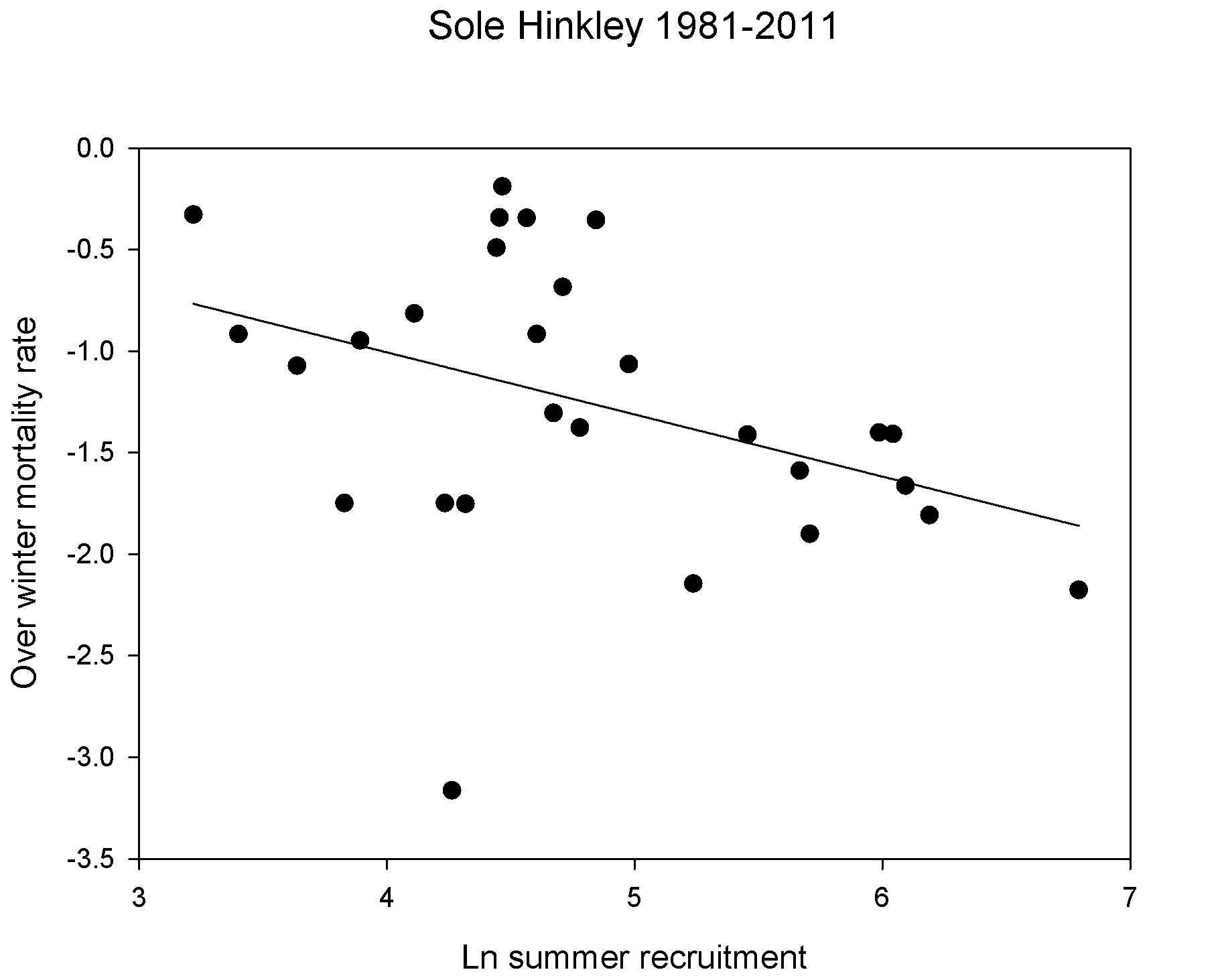


Figure 28 The relationship between overwintering mortality rate and recruitment for sole, *Solea solea* in Bridgwater Bay

## Sprat, *Sprattus sprattus*

Because of a lack of sampling in autumn 1986 the time series was analysed from 1987 onwards. Sprat is one of the most abundant species in Bridgwater Bay and this allowed a more detailed analysis of the age structure and temporal variation in recruitment than was possible for many species. Of particular importance is the fact that there is a migration of new recruits in the summer and a second wave of arrivals of adult fish during the winter.

### Relationship between log population change and log population size

The slope of log change in population size (Figure 29) offers no proof for density-dependence. The log change in populations log population plot shows a negative slope of -0.4 which is insufficiently steep to prove density-dependence, but it is consistent with a density dependent model.

There is also no evidence of a non-linear relationship or a break-point indicating a threshold response.

**The relationship between Log Population Change and Log Population size gives no support for the existence of density-dependence.**


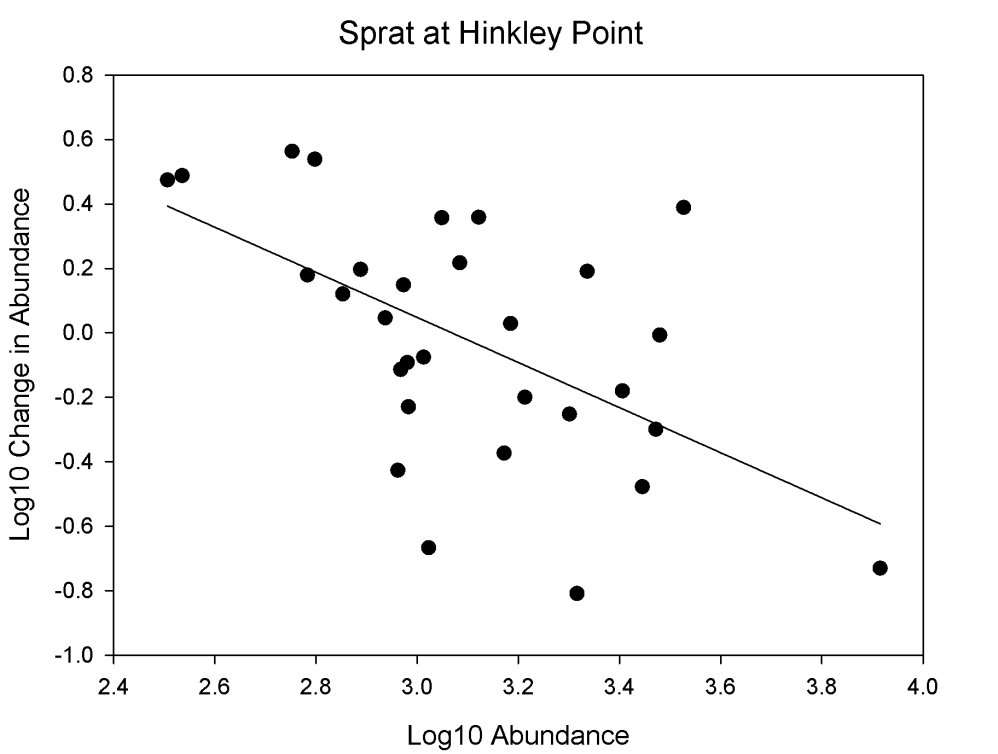


Figure 29 The relationship between population size and population change for sprat, Sprattus sprattus.

### Bulmer’s test

Using count data from 1987 to 2013 inclusive the results for Bulmer’s tests were as follows:

R = 0.68, R_0.5_ = 1.165, **significant for density dependence at 5% level.**

R^*^= -0.099, R_0.5_ = -0.348, **non-significant for density dependence at 5% level.**

### Additional evidence

To show the temporal trend in sprat abundance two abundance indices were calculated and plotted in Figure 30. The total abundance index is the sum of the number of sprats of all age groups caught in the 12 regular monthly samples for each year. No total abundance index is available for 1986 as only 8 monthly samples were collected. The recruitment index is the sum of O-group individuals caught in the monthly samples from June to September inclusive. The indices are plotted on a log10 scale to visualize long-term trend. Both indices show considerable between-year variation, but exhibit no long-term trend. The coefficient of variation of the recruitment index is 87.7% compared with 97.1% for the total index.

General Linear Modelling was used to identify potential explanatory variables for the between year variation in recruitment. All of the variables presented in **Table 1** were initially considered. Both Recruitment and adult abundances were log transformed to reduce their highly skewed distributions. By a process of variable selection and elimination the best model is represented by the word equation:

Recruitment_i+1_= Adult Abundance year_i_ + sunspot number_i_ + NAOI(Jan-July)_i+1_ + salinity_i_.

The analysis of variance table for this model is given in **Table 2**. This analysis showed that recruitment declined with increasing adult abundance and average salinity. Conversely, recruitment increased with sunspot number and as the NAOI became more negative.

The negative relationship between recruitment and adult abundance is indicative of density-dependence. Using the data from the regular monthly surveys a stock-recruitment relationship was generated. The abundance of newly recruited fish was estimated by summing the number caught June to September inclusive. The spawning stock size was estimated as the sum of the fish present for the months of November to February inclusive as these fish would spawn the following spring and early summer. There is a negative correlation between stock size and recruitment (Spearman Rank Correlation =-0.324, n=29, P>0.05) which is not significant at the 5% level and the peak recruitment occurs at intermediate adult abundance. Given these observations and the fact that sprats are known to eat their own eggs (Koster & Mollmann, 2000) a Ricker curve:

$$R=aSe^{-bS}$$

where R is the number of recruits, S is the spawning stock size and a and b constants was fitted to these data (Figure 31). The observed stock-recruitment relationship shows considerable variability around the fitted stock-recruitment relationship which potentially, in part, could be explained by the role of the variables for sunspot number, salinity and NAOI which had previously been shown to be correlated with recruitment success. The search for the most parsimonious model combining the stock-recruitment relationship and these physical factors identified the expression:

Recruitment i+1 = 961+2.97Se-0.00423S + Sunspotsi - 35.2 x Salinityi-57.9 x NAOI(Jan-July) i+1, (R2 = 0.477).

This expression reflects the fact that recruitment is greatest at intermediate stock levels, when sunspot number is high, salinity lower than average and the NAOI is negative. The predicted recruitment is shown as a red line in Figure 30. This model gives a generally good fit to the data although it exaggerated the low recruitment observed in 2009.


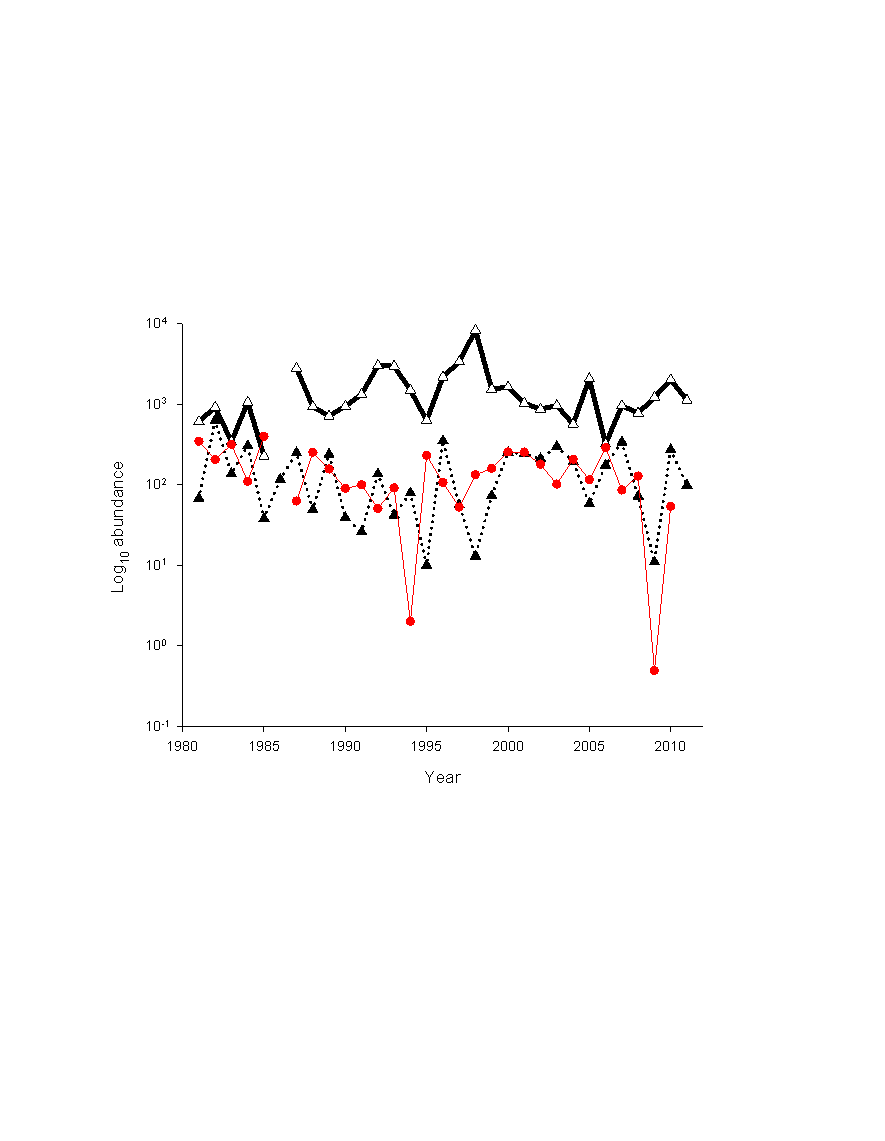


Figure 30 The change in the rate of capture of sprats at Hinkley Point Power Station since 1981. The black upper thicker line is total abundance of all age classes caught per year. The lower black dotted line is O-group abundance. The red line marks the predicted recruitment using a model combining a stock-recruitment relationship with the effects of sunspot number, salinity and the North Atlantic Oscillation.


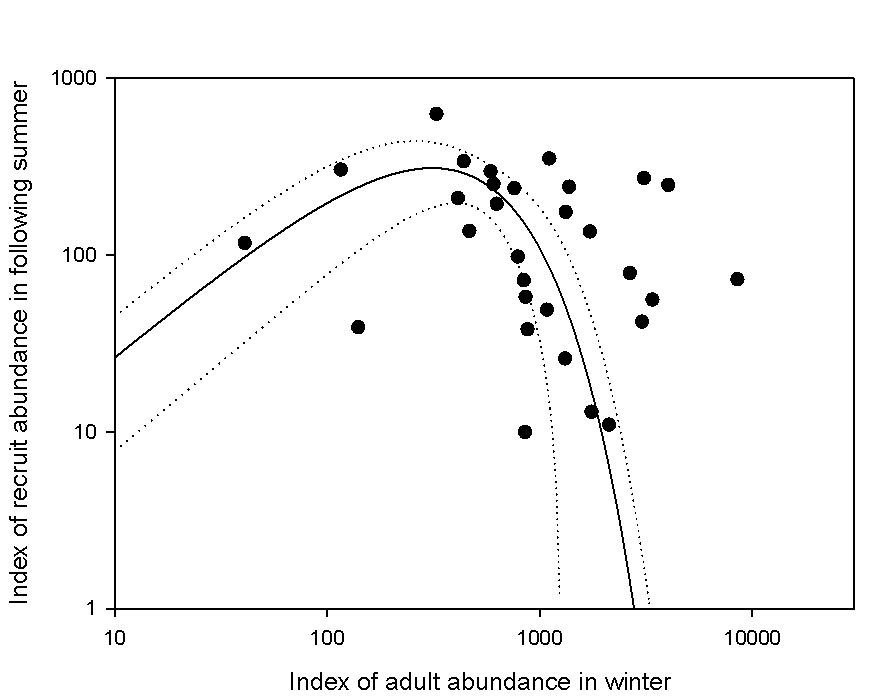


Figure 31 The relationship between adult abundance during the winter (November to February) and recruitment the following summer (June to September). A Ricker curve has been fitted to the data using non-linear regression. The upper and lower 95 percentiles of this curve are shown as dotted lines.

Table 1 A summary and source of the physical variables examined as potential predictors of sprat growth and recruitment.

| **Variable** | **Source** |  |
| --- | --- | --- |
| Salinity | Monthly recordings taken at the time of sampling. |  |
| Seawater temperature | Monthly recordings taken at the time of sampling |  |
| Freshwater flow | Monthly flow measured at the Bewdley on the River Severn downloaded from the Environmental Agency http://www.nwl.ac.uk/ih/nrfa/webdata/054001/g.html |  |
| Air temperature- | Average for Southern English sites based on daily records. Downloaded from http://www.metoffice.gov.uk/climate/uk/datasets/ | |
| Sunspot number | Average annual value obtained from SIDAC at http://sidc.oma.be/sunspot-data/ | |
| NAOI Annual average | The difference between the normalised sea level pressure over Gibraltar and the normalised sea level pressure over Southwest Iceland is used as an index of the North Atlantic Oscillation. The average of the 12 monthly values is used. The data used here were downloaded from http://www.cru.uea.ac.uk/cru/data/nao/ (up to 1999) and http://www.cru.uea.ac.uk/~timo/datapages/naoi.htm (from 2000 onwards) | |
| NAOI Winter average | The difference between the normalised sea level pressure over Gibraltar and the normalised sea level pressure over Southwest Iceland is used as an index of the North Atlantic Oscillation. The average of the months December to march is used. The data used here were downloaded from http://www.cru.uea.ac.uk/cru/data/nao/ (up to 1999) and http://www.cru.uea.ac.uk/~timo/datapages/naoi.htm (from 2000 onwards) | |
| Position of the north wall of the Gulf Stream | Monthly Means of the 1st Principal Component of the position of the North Wall of the Gulf Stream 1966-2010 were obtained from http://www.pml-gulfstream.org.uk/ | |
| Rainfall | Average of monthly total for Southern Britain from many recording sites. Downloaded from http://www.metoffice.gov.uk/climate/uk/datasets/ | |
| Sunshine hours | Monthly totals for Southern English sites based on daily records. Downloaded from http://www.metoffice.gov.uk/climate/uk/datasets/ | |

Table 2 Analysis of variance table for the variables included in the final predictive model for sprat recruitment generated using General Linear modelling.

| Source | DF1 | DF2 | Inc. SS | Adj. SS | MS | F | Probability |
| --- | --- | --- | --- | --- | --- | --- | --- |
| Adult Abundance | 1 | 24 | 4.02832 | 3.72128 | 3.72128 | 4.39386 | 0.0467916 |
| Sunspot number | 1 | 24 | 1.62456 | 4.24529 | 4.24529 | 5.01257 | 0.0347001 |
| NAOI(Jan-July) | 1 | 24 | 4.08439 | 5.43011 | 5.43011 | 6.41154 | 0.0182954 |
| Average Salinity | 1 | 24 | 3.85524 | 3.85524 | 3.85524 | 4.55202 | 0.0433034 |
| Error | 24 |  | 20.3263 |  | 0.846928 |  |  |
| Total | 28 |  | 33.9188 |  |  |  |  |

## Thin-lipped Grey Mullet, *Liza ramada*

This is a winter migrant and because some monthly samples were missing in winter 1986 time series analysis used data from 1987 onwards.

### Relationship between log population change and log population size

The slope of log change in population size offers no proof for density-dependence. The log change in populations log population plot shows a negative slope of -0.7 which is insufficiently steep to prove DD. But it is consistent with a density dependent model.

There is also no evidence of a non-linear relationship or a break-point indicating a threshold response.

**The relationship between Log Population Change and Log Population size gives no support for the existence of density-dependence.**


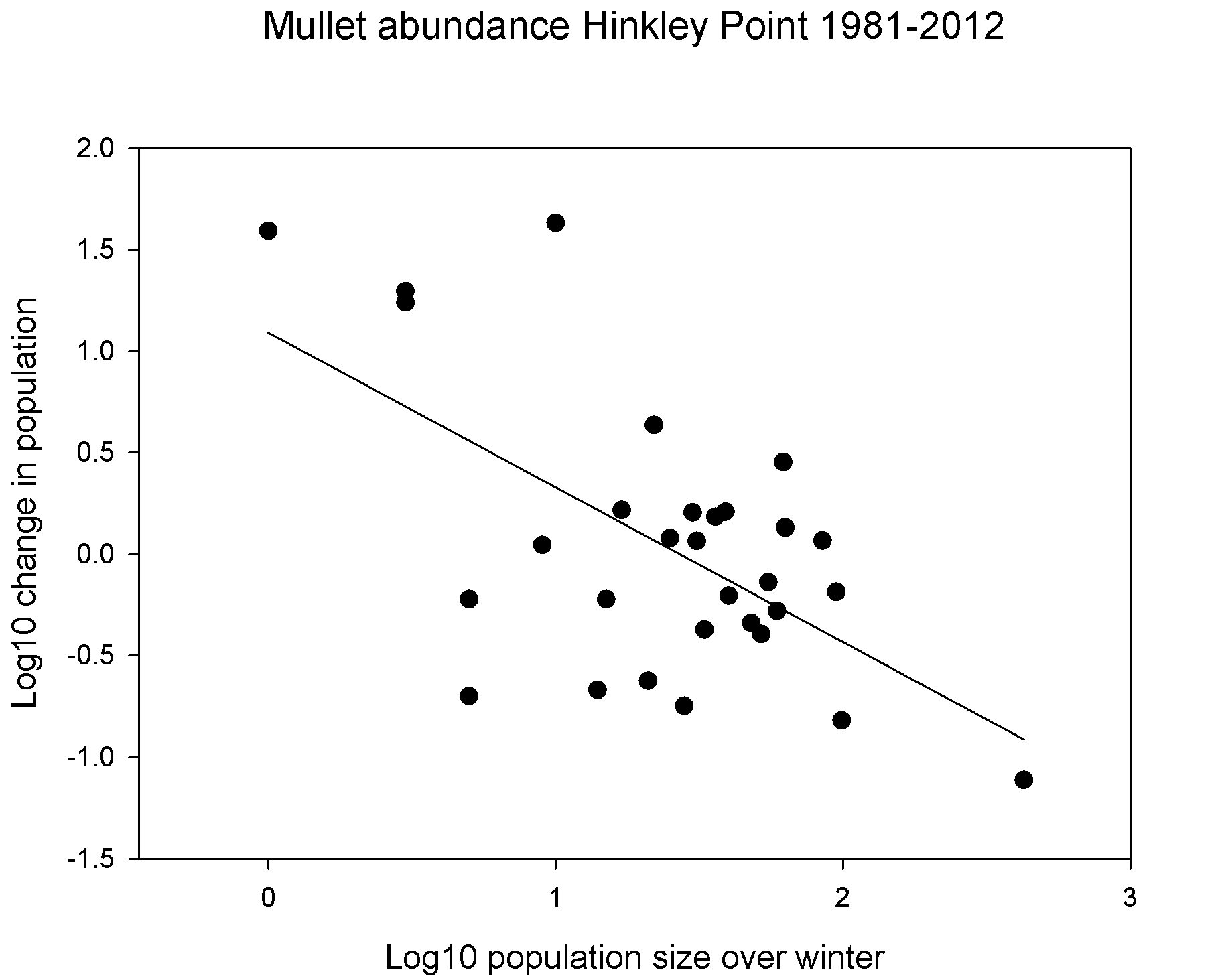


Figure 32 The relationship between the annual winter abundance of mullet and the change in abundance for Bridgwater Bay between 1981 and 2012. Data for 1986 is excluded.

### Bulmer’s test

Using data from 1987 to 2013 inclusive the results for Bulmer’s tests were as follows:

R = 0.67, R_0.5_ = 1.165, **significant for density dependence at 5% level.**

R^*^= -0.552, R_0.5_ = -0.348, **significant for density dependence at 5% level.**

### Additional information

The first point to note about mullet is that they arrive in winter and the population comprises many year classes. As we have a mixed age group population it is best to consider biomass rather than number.

Mullet in Bridgwater Bay comprise a number of age classes and count abundance varies to a greater extent than biomass because of the between year variation in recruitment. Figure 33 shows that the biomass maximum during the winter is notably stable.


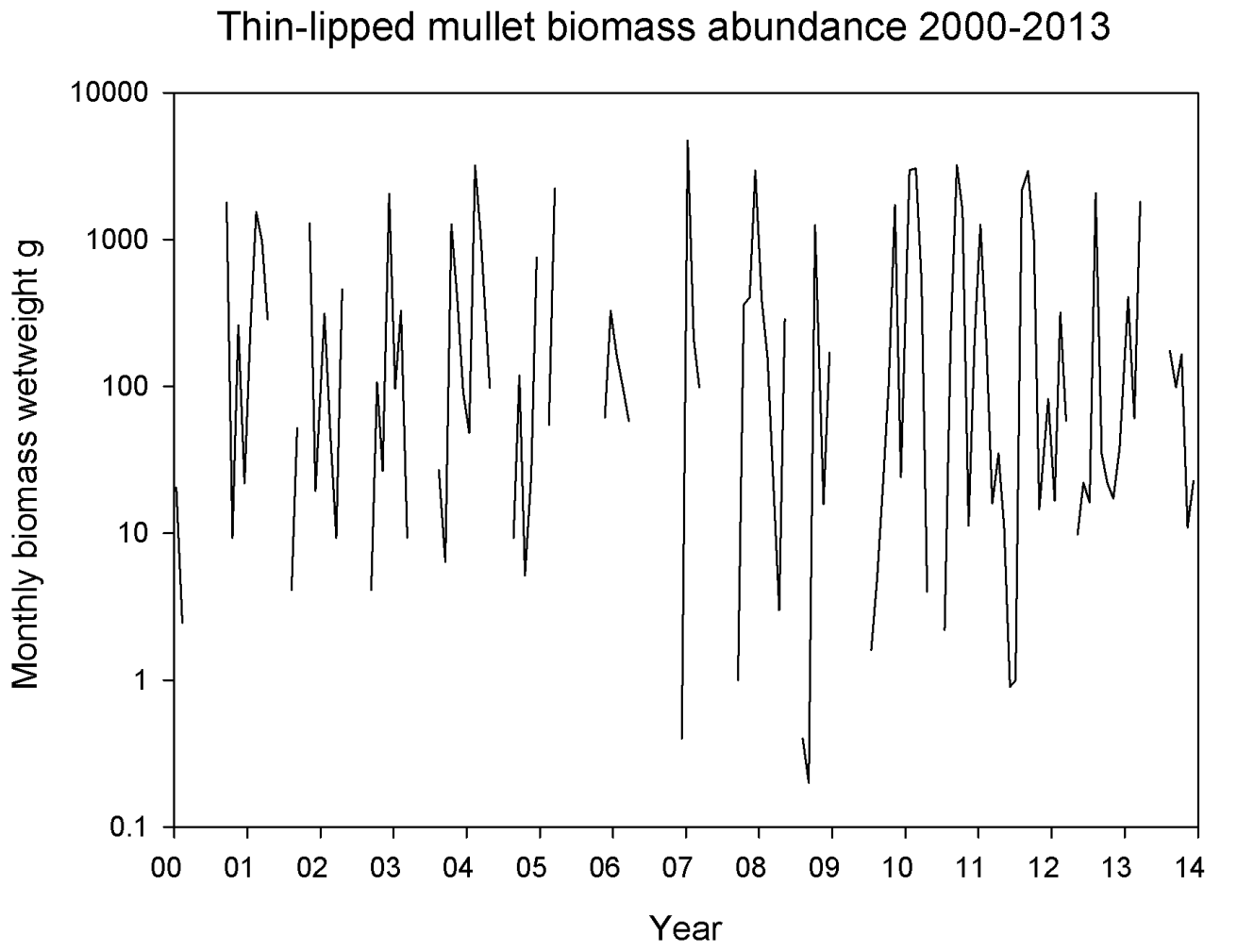


Figure 33 Temporal variation in mullet, *Liza ramada*, biomass from monthly samples collected between 2000 and 2013 in Bridgwater Bay

## Transparent goby, *Aphia minuta*

This is a small planktonic goby with a life span of approximately 1 year at most. While never abundant it is notably consistent in its presence and has been observed every year since 1981.

### Relationship between log population change and log population size

The slope of log change in population size offers no proof for density-dependence (Figure 34). The log change in populations log population plot shows a negative slope of -1.023 which is insufficiently steep to prove density-dependence, but it is consistent with a density dependent model.

However, there was a sharp change in transparent goby abundance in the mid 1980s probably linked to a major change in plankton community structure reported by a number of observers. The data post 1987 was therefore analysed separately. The slope of log change in population size from 1988-2013 offers evidence for density-dependence (Figure 34). The log change in populations – log abundance plot shows a negative slope of -1.18 which is sufficiently greater than -1 to be indicative of density-dependence.

There is no evidence of a non-linear relationship or a break-point indicating a threshold response.

**The relationship between Log Population Change and Log Population size gives support for the existence of density-dependence.**


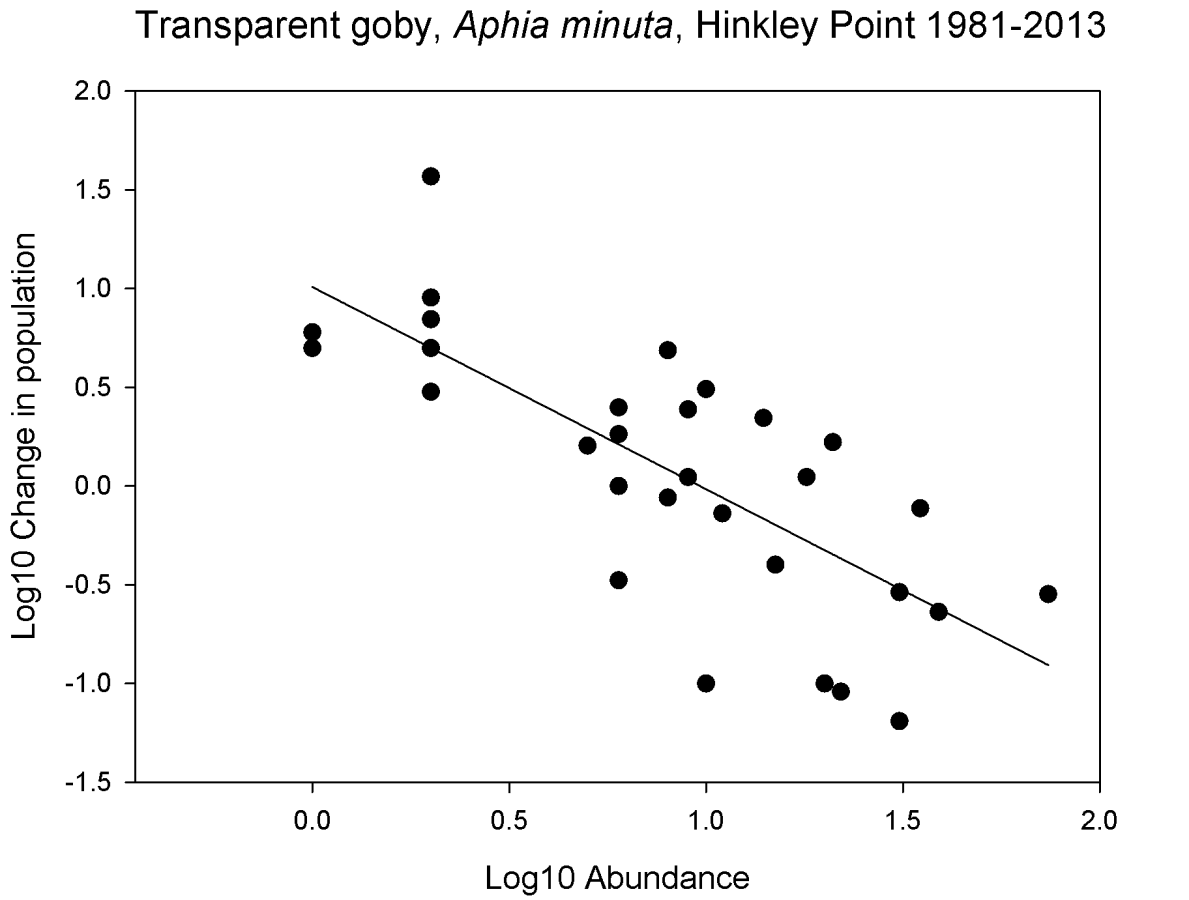


Figure 34 The relationship between population size and change for transparent goby*, Aphia minuta*, at Hinkley Point. This plot includes all years 1981 to 2013.


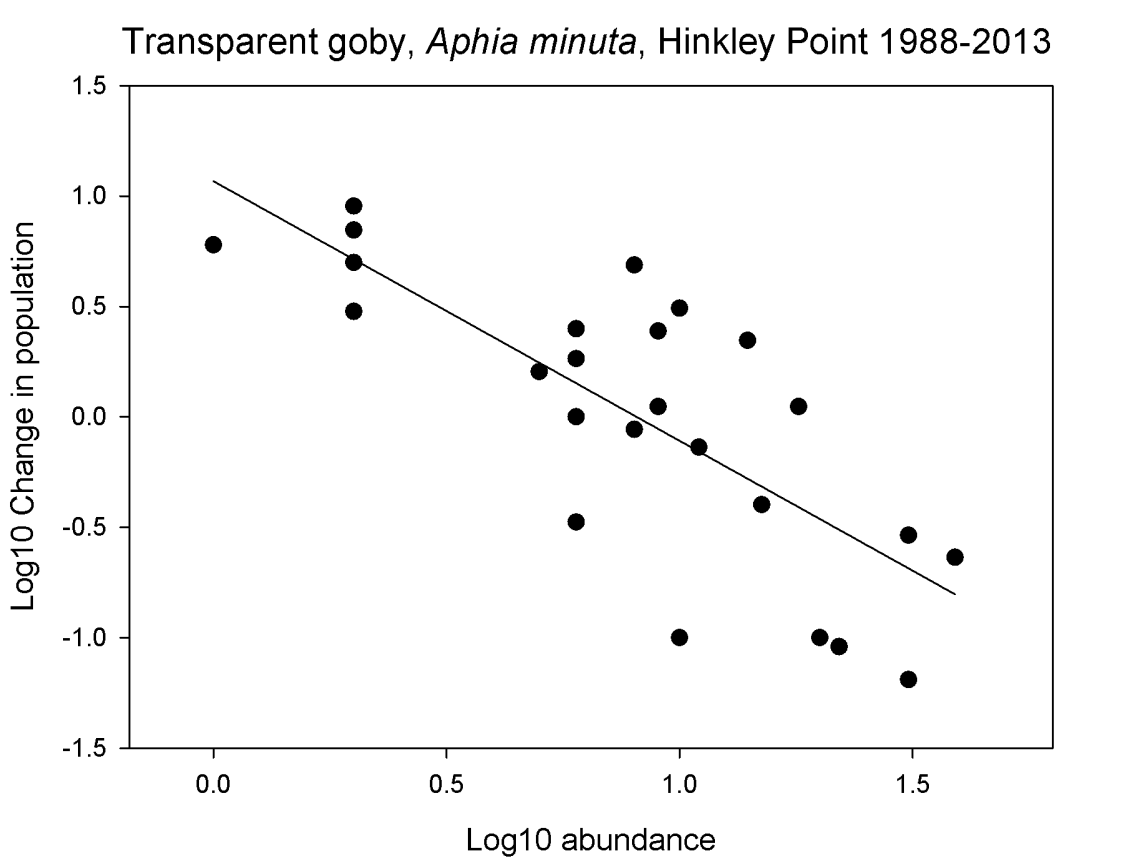


Figure 35 The relationship between population size and change for transparent goby*, Aphia minuta*, at Hinkley Point. This plot includes all years 1988 to 2013.

### Bulmer’s test

Using data from 1987 to 2013 inclusive the results for Bulmer’s tests were as follows:

R = 0.48, R_0.5_ = 1.165, **significant for density dependence at 5% level.**

R^*^= -0.16, R_0.5_ = -0.348, **non-significant for density dependence at 5% level.**

## Whiting, *Merlangius merlangus*

This is one of the most abundant fish in Bridgwater Bay.

### Relationship between log population change and log population size

Preliminary analysis of the relationship between log population change and log population size indicated that the relationship was non-linear (Figure 36 and Figure 37) and could be described by a rational equation of the form :

$Y=(a-bX)/(1+cX+dX^{2})$


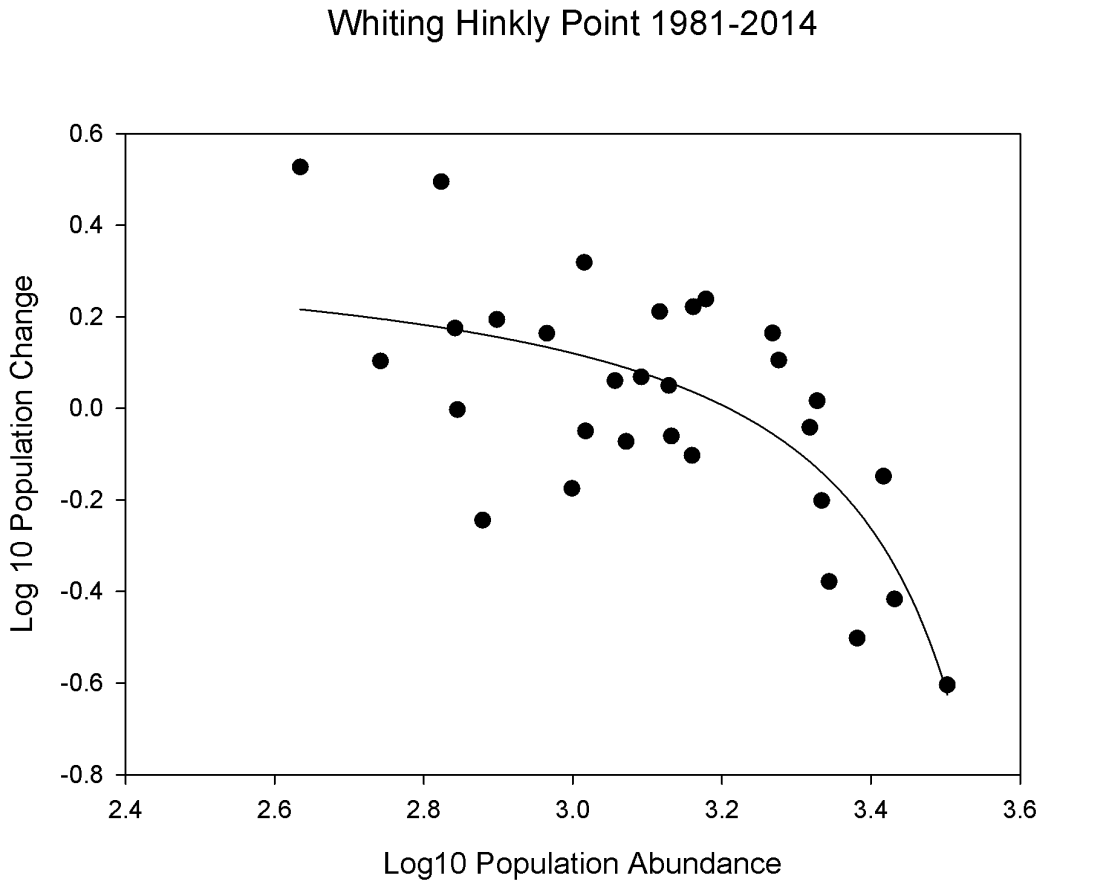


Figure 36 The relationship between population size and change for the whiting Merlangius merlangius at Hinkley Point. The rational curve was fitted by regression using a best fit algorithm.

The important point to note is not the particular curve chosen to fit the data, but the fact that it is either non-linear or possesses a threshold when the relationship abruptly changes. Either of these features provides evidence for density-dependence.

To test for the potential existence of a threshold at about log_10_ abundance of 3.3 the Chow test for structural breaks was undertaken. This gave a test statistic of 4.36 and a probability of 0.023 indicating that the null hypothesis that the two regressions either side of the break are the same should be rejected. Figure 37 shows the fit of a single linear regression and also the two regressions either side of the break point.


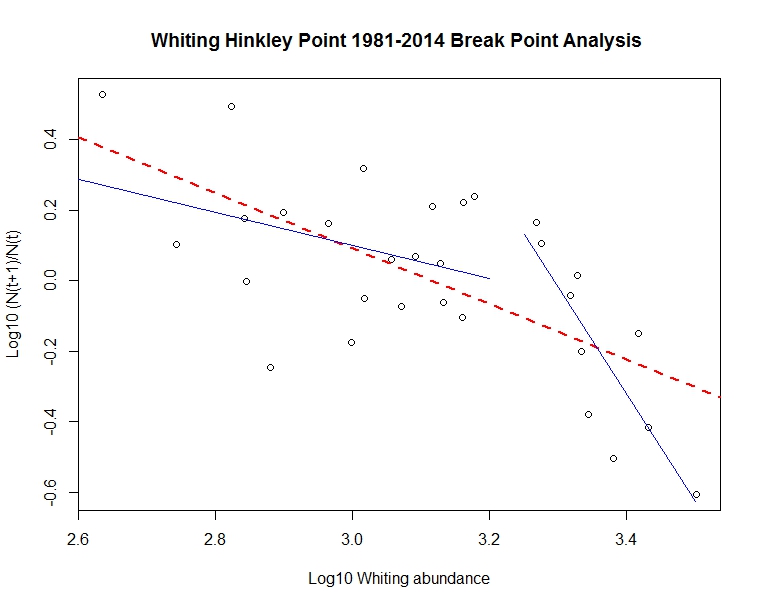


Figure 37 The fitted linear regressions either side of a break point at x=3.3.

Analysis of the log population change -log population size relationship indicates non-linearity and therefore density-dependence.

**The relationship between Log Population Change and Log Population size gives support for the existence of density-dependence.**

### Bulmer’s tests

Using count data from 1987 to 2013 inclusive the results for Bulmer’s tests were as follows:

R = 0.575, R_0.5_ = 1.16, **significant for density dependence at 5% level.**

R^*^= -0.28, R_0.5_ = -0.348, **non-significant for density dependence at 5% level.**

## Less common species showing a random pattern of occurrence

There is a small number of frequently observed (on an annual basis) species that are never abundant and they are approximately random in their occurrence. Their observed frequency distributions of annual abundance typically fit a Poisson distribution. Some may fit a more complex abundance model as they live in shoals. It is therefore likely that occurrence through time is random, but the number observed is clumped. The number of observations is too few to allow model fitting. A key characteristic of these species is that they favour habitats which are not found within Bridgwater Bay. For example the 3-spined stickleback is a freshwater and upper estuarine species that is only caught when the rivers are in flood and it is washed into the lower estuary; the sandeel is a species of clean sand and not mud or muddy sand as found in Bridgwater Bay.

Species whose occurrence and abundance is unpredictable and are only ever present in small numbers are as follows:

Horse mackerel, *Trachurus trachurus*

Gurnard, *Trigla lucerna*

Nilsson’s pipefish, *Sygnathus rostellatus*

Greater pipefish, *Sygnathus acus*

Black seabream, *Spondyliosoma cantharus*

15-spined stickleback, *Spinachia spinachia*

Sandeel, *Ammodytes tobianus*

Dragonette, *Callionymus lyra*

Thornback ray, *Raja clavata*

None of the above species can exhibit density-dependent control in Bridgwater Bay as their populations lie outside the region and their occurrence is linked to physical factors such as storms, tides and floods.

## References

1. Freckleton R.P., Watkinson A.R., Green R.E., Sutherland W.J. 2006 Census error and the detection of density dependence. *Journal of Animal Ecology* **75**(4), 837-851.

2. Henderson P.A., Holmes R.H.A. 1990 Population stability over a ten-year period in the short-lived fish *Liparis liparis* (L.). *Journal of Fish Biology* **37**, 605-616.

3. Bulmer M.G. 1975 The statistical analysis of density dependence. *Biometrics* **31**, 901-911.

4. Henderson P.A., Corps M. 1997 The role of temperature and cannibalism in interannual recruitment variation of bass in British waters. *J Fish Biol* **50**, 280-295.

5. Henderson P.A., Seaby R.M.H. 1999 Population stability of the sea snail at the southern edge of its range. *J Fish Biol* **54**, 1161-1176.

# Appendix 1 R code for testing Chow statistic for break point

#Read in the data from the file

whiting <- read.table("C:/Users/Peter/Dropbox/R working/Whiting DD data.csv", header=TRUE,

sep=",")

#Pint out the data to check it is OK

summary

whiting

## Run three regressions (1 unrestricted, 2 restricted to above and below break)

r.reg = lm(logchange ~ logN, data = whiting)

ur.reg1 = lm(logchange ~ logN, data = whiting[whiting$logN > 3.2,])

ur.reg2 = lm(logchange ~ logN, data = whiting[whiting$logN < 3.2,])

# review the regression results

summary(r.reg)

summary(ur.reg1)

summary(ur.reg2)

## Calculate sum of squared residuals for each regression

SSR = NULL

SSR$r = r.reg$residuals^2

SSR$ur1 = ur.reg1$residuals^2

SSR$ur2 = ur.reg2$residuals^2

# K is the number of regressors in our model

K = r.reg$rank

# Computing the Chow test statistic (F-test)

numerator = ( sum(SSR$r) - (sum(SSR$ur1) + sum(SSR$ur2)) ) / K

denominator = (sum(SSR$ur1) + sum(SSR$ur2)) / (nrow(whiting) - 2*K)

chow = numerator / denominator

chow

# Calculate P-value

1-pf(chow, K, (nrow(whiting) - 2*K))

# Plot the results

plot(whiting,main="")

# restricted model

abline(r.reg, col = "red",lwd = 2, lty = "dashed")

# restricted model 1

segments(0, ur.reg2$coefficients[1], 3.2,

ur.reg2$coefficients[1]+3.2*ur.reg2$coefficients[2], col= 'blue')

# restricted model 2

segments(3.25, ur.reg1$coefficients[1]+3.25*ur.reg1$coefficients[2],

3.5, ur.reg1$coefficients[1]+3.5*ur.reg1$coefficients[2], col= 'blue')
